# Circulating miRNAs as Potential Biomarkers for Celiac Disease Development

**DOI:** 10.3389/fimmu.2021.734763

**Published:** 2021-12-07

**Authors:** Ineke L. Tan, Rodrigo Coutinho de Almeida, Rutger Modderman, Anna Stachurska, Jackie Dekens, Donatella Barisani, Caroline R. Meijer, María Roca, Eva Martinez-Ojinaga, Raanan Shamir, Renata Auricchio, Ilma R. Korponay-Szabó, Gemma Castillejo, Hania Szajewska, Sibylle Koletzko, Alexandra Zhernakova, Vinod Kumar, Yang Li, Marijn C. Visschedijk, Rinse K. Weersma, Riccardo Troncone, M. Luisa Mearin, Cisca Wijmenga, Iris Jonkers, Sebo Withoff

**Affiliations:** ^1^ Department of Genetics, University of Groningen and University Medical Center Groningen, Groningen, Netherlands; ^2^ Department of Gastroenterology and Hepatology, University of Groningen and University Medical Center Groningen, Groningen, Netherlands; ^3^ Department of Biomedical Data Sciences, Section Molecular Epidemiology, Leiden University Medical Center, Leiden, Netherlands; ^4^ Center of Development and Innovation, University of Groningen and University Medical Center Groningen, Groningen, Netherlands; ^5^ School of Medicine and Surgery, University of Milano-Bicocca, Monza, Italy; ^6^ Department of Pediatrics, Leiden University Medical Center, Leiden, Netherlands; ^7^ Celiac Disease and Digestive Immunopathology Unit, Instituto de Investigación Sanitaria La Fe, La Fe University Hospital, Valencia, Spain; ^8^ Celiac Disease and Digestive Immunopathology Unit, Instituto de Investigación Sanitaria La Fe, La Fe University Hospital, Madrid, Spain; ^9^ Institute of Pediatric Gastroenterology, Nutrition, and Liver Diseases, Schneider Children’s Medical Center, Petach Tikva, Israel; ^10^ Sackler Faculty of Medicine, Tel Aviv University, Tel Aviv, Israel; ^11^ Department of Medical Translational Sciences and European Laboratory for the Investigation of Food Induced Diseases, University Federico II, Naples, Italy; ^12^ Coeliac Disease Center, Heim Pál National Paediatric Institute, Budapest, Hungary and Dept. of Paediatrics, Faculty of Medicine and Clinical Center, University of Debrecen, Debrecen, Hungary; ^13^ Unitat de gastroenterologia pediàtrica, Hospital Universitari Sant Joan de Reus, Universitat Rovira i virgili, Institut d'Investigació Sanitària Pere Virgili (IISPV), Reus, Spain; ^14^ Department of Paediatrics, The Medical University of Warsaw, Warsaw, Poland; ^15^ Department of Pediatrics, Dr. von Hauner Children’s Hospital, Ludwig-Maximilians-Universität München (LMU) Klinikum Munich, Munich, Germany; ^16^ Department of Pediatric Gastroenterology and Nutrition, School of Medicine Collegium Medicum University of Warmia and Mazury, Olsztyn, Poland; ^17^ Department of Internal Medicine and Radboud Center for Infectious Diseases (RCI), Radboud University Medical Center, Nijmegen, Netherlands; ^18^ Department of Computational Biology for Individualised Infection Medicine, Centre for Individualised Infection Medicine (CiiM) & TWINCORE, Joint Ventures Between the Helmholtz-Centre for Infection Research (HZI) and the Hannover Medical School (MHH), Hannover, Germany; ^19^ Department of Internal Medicine and Radboud Institute for Molecular Life Sciences, Radboud University Medical Center, Nijmegen, Netherlands

**Keywords:** small RNA sequencing, pre-diagnostic marker, pre-clinical marker, autoimmunity, celiac disease

## Abstract

**Background & Aims:**

Celiac disease (CeD), an immune-mediated disease with enteropathy triggered by gluten, affects ~1% of the general European population. Currently, there are no biomarkers to predict CeD development. MicroRNAs (miRNAs) are short RNAs involved in post-transcriptional gene regulation, and certain disease- and stage-specific miRNA profiles have been found previously. We aimed to investigate whether circulating miRNAs can predict the development of CeD.

**Methods:**

Using next-generation miRNA-sequencing, we determined miRNAs in >200 serum samples from 53 participants of the PreventCD study, of whom 33 developed CeD during follow-up. Following study inclusion at 3 months of age, samples were drawn at predefined ages, diagnosis (first anti-transglutaminase antibody (TGA) positivity or diagnostic biopsy) and after the start of a gluten-free diet (GFD). This allowed identification of circulating miRNAs that are deregulated before TGA positivity. For validation of the biomarkers for CeD and GFD response, two additional cohorts were included in subsequent meta-analyses. Additionally, miRNAs were measured in duodenal biopsies in a case-control cohort.

**Results:**

53 circulating miRNAs were increased (27) or decreased (26) in CeD *versus* controls. We assessed specific trends in these individual miRNAs in the PreventCD cohort by grouping the pre-diagnostic samples of the CeD patients (all had negative TGA) by how close to seroconversion (first sample positive TGA) the samples were taken. 8/53 miRNAs differed significantly between controls and samples taken <1 year before TGA positivity: miR-21-3p, miR-374a-5p, 144-3p, miR-500a-3p, miR-486-3p let-7d-3p, let-7e-5p and miR-3605-3p. 6/26 downregulated miRNAs reconstituted upon GFD, including miR-150-5p/-3p, whereas no upregulated miRNAs were downregulated upon GFD. 15/53 biomarker candidates also differed between CeD biopsies and controls, with a concordant direction, indicating that these circulating miRNAs might originate from the intestine.

**Conclusions:**

We identified 53 circulating miRNAs that are potential early biomarkers for CeD, of which several can be detected more than a year before TGA positivity and some start to normalize upon GFD.

## Introduction

In celiac disease (CeD), genetically susceptible individuals develop a small intestinal immune response to gluten, a group of storage proteins present in food items containing wheat, rye or barley (1). Partially degraded gluten proteins pass the small-intestinal epithelial barrier and are deamidated by the enzyme transglutaminase 2 (TG2). Specific deamidated gluten peptides bind strongly to HLA-DQ2 or -DQ8, resulting in activation of gluten-specific CD4+ T cells, which then initiate an immune response by secreting cytokines that activate CD8+ T cells (2, 3). The activated CD8+ T cells that migrate to the epithelial layer (called intra-epithelial lymphocytes) and are then “licensed to kill” epithelial barrier cells, resulting in villous atrophy (4). Simultaneously, B cells interact with activated gluten-specific CD4+ T cells and secrete disease-specific autoantibodies against TG2 (TGA), of which the detection is the current mainstay of CeD diagnosis (3). The only current treatment for CeD is a strict lifelong gluten-free diet (GFD).

Epidemiological studies based on screening for TGA seroprevalence suggest that approximately 1-2% of the Caucasian population has CeD, but that at least half of the individuals with CeD remain undiagnosed (4, 5). The age of CeD diagnosis ranges from the first encounter with gluten in the first year, too late in life. Moreover, CeD is characterized by a wide array of symptoms varying from gastrointestinal symptoms (abdominal pain, bloating, chronic diarrhea, constipation) and/or extra-intestinal symptoms (e.g. iron-deficiency anemia, fatigue, poor growth in children, weight loss), and many persons with CeD have no signs and symptoms at all. Altogether, these features make it difficult to diagnose CeD (2, 6–8). Untreated CeD may aggravate symptoms (e.g. weight loss, failure to thrive in children, moodiness and loss of energy) and CeD-associated complications (e.g. osteoporosis) that decrease quality of life (9–12). The importance of early diagnosis for avoiding symptoms and complications underlines the need for tools that can detect CeD as early as possible, ideally before disease onset and accompanying symptoms.

Historically, the ‘gold standard’ for diagnosing CeD was the histopathological detection of villous atrophy and increased numbers of intra-epithelial lymphocytes in duodenal biopsies collected by upper endoscopy. However, these lesions are not specific for CeD. In the last few decades, increased TGA and anti-endomysium autoantibody concentrations in serum have been added to the diagnostic work-up and have been used for screening of persons at risk for CeD (2, 3, 13). The major drawback of these antibody-based tests is that they cannot be used as predictive markers of disease development because in the majority of patients these antibodies are found elevated when intestinal mucosal lesions are already present (3, 14–16). For early detection of CeD, preferably before the onset of intestinal damage, it would be valuable to identify novel biomarkers for CeD development. Ideally, these biomarkers would be blood-based, detectable at an early stage of CeD onset and able to monitor GFD adherence.

Circulating microRNAs (miRNAs) represent such biomarker candidates. These small non-coding RNAs (19-24 nucleotides) appear to be stable in the extracellular environment in different biofluids, including blood, and specific circulating miRNAs have been shown to be detectable in blood in a disease- or even disease stage-specific fashion (17–21). In previous studies applying array-based approaches, CeD-specific miRNA profile changes were observed in small intestinal biopsies of CeD patients (22–24). Some of the deregulated miRNAs were also later detected in the circulation of CeD patients at the time of diagnosis (25).

We applied a next-generation miRNA-sequencing approach to profile extracellular/circulating miRNAs. The advantage of the next generation sequencing approach is that it is not limited by an array-design nor dependent on PCR-primer sets, thus allowing for holistic screening of the entire miRNA repertoire catalogued in the current version of miRbase (26). To find biomarkers, we used three different studies, including the longitudinal prospective CeD birth cohort, PreventCD (15). Participants of PreventCD are at high risk of developing CeD because they carry the HLA-risk alleles and have at least one 1^st^ degree family member diagnosed with CeD. They were enrolled at birth and were followed up to 12 years of age. The availability of longitudinal samples from birth for both participants who did develop CeD and those who did not, enabled us to search for CeD biomarkers that arise before celiac-specific autoantibodies (TGA) are increased in serum.

Altogether, we detected 53 miRNAs in circulation that are potential early biomarkers for CeD. Changes in several of these miRNAs were detectable in blood more than two years before CeD diagnosis by TGA antibody detection and small bowel biopsies, and six of them began to normalize once the participant started treatment with a GFD. We therefore propose that these miRNAs represent novel biomarker candidates for early detection of CeD.

## Material and Methods

### Sample Collection

Serum samples of the PreventCD cohort collected in the context of a prospective, multicenter study were used to generate the explorative dataset. In short, infants at high risk of developing CeD were included after birth and followed up prospectively (15, 27). Circulating microRNA (here defined as all extracellular miRNAs present in the circulation, which includes exosomic miRNAs and miRNAs potentially present in other extracellular vesicles or in protein-miRNA aggregates) profiles were generated from 250 serial serum samples obtained from 53 participants of whom 33 developed CeD during the course of the study (**Table 1** shows the number of samples included in the final analyses after the quality control; **Supplementary Table S4** shows the number of samples excluded in the quality control). The remaining 20 individuals who did not develop CeD within the timeframe of the PreventCD study provided the control samples. Samples were drawn at 4, 6, 9, 12, 18 or 24 months of age, at time of CeD diagnosis (taken at first positive TGA sample or at the diagnostic biopsy). The samples included in this “Diagnosis” group, were taken on average 1.71 months after seroconversion (first positive TGA sample). Additional samples were included after start of a GFD (median: 7.4 months after start of the GFD, range: 2.3‒40 months). Serum TGA levels were determined at each timepoint by the Celikey™ Varelisa ELISA or ELIA assays, where positivity was assigned to results above 6 U/ml or 7 U/ml, respectively.

**Table 1:** Overview of samples.

Controls					CeD Patients						Healthy Volunteers	
Non-CeD	High-risk CeD				Before Diagnosis				At Diagnosis	On GFD	On GFD	Off GFD
	**M4**	M6-9	M12	M18-24	**M4**	M6-9	M12	M18-24				
	13	20	17	18	19	22	23	24	21	29		
9*									33*	10		
											12	12

This overview shows how many circulating microRNA samples were included in the final analyses. M4-M24: months of age. In the CeD patients of the PreventCD cohort, the first sample showing positive IgA anti-transglutaminase antibodies (at seroconversion) or samples close to the diagnostic biopsy were grouped in the “At diagnosis” group. All samples of PreventCD CeD patients taken prior to seroconversion, with negative IgA anti-transglutaminase antibodies, were grouped in the “Before Diagnosis group”. *In the Milano-Bicocca cohort intestinal microRNA profiles were generated from duodenal biopsies from 10 controls (all control samples in the biopsy group passed quality control) and 33 patients at diagnosis.

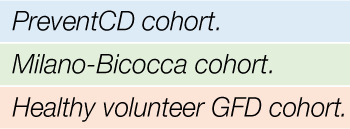

Additionally, samples were derived from an independent case-control cohort consisting of patients included in the University Medical Hospital of Milano-Bicocca, Monza, Italy (**Table 1** shows the number of samples included in the final analyses after the quality control; **Supplementary Table S4** shows the number of samples excluded in the quality control) (discussed as the ‘Milano-Bicocca cohort’). In this cohort, plasma samples were collected from 33 pediatric CeD patients at time of diagnosis and from 10 of these patients 2 years after start of the GFD. Control plasma samples were obtained from 10 pediatric patients in whom CeD was excluded by histopathological examination of small-intestinal biopsies. For all Milano-Bicocca subjects (both CeD patients and controls), we also had biopsy-derived RNA taken at the time of plasma collection (time of diagnosis). Additional clinical characteristics of participants of the PreventCD and the Milano-Bicocca cohorts are presented in the **Supplementary Materials** and **Supplementary Tables S1**–**3**.

We also aimed to investigate the effect of GFD on circulating miRNA profiles. For this analysis, we used the GFD samples available from the PreventCD and the Milano-Bicocca cohorts but also included samples from a healthy adult cohort of 12 healthy adults without self-reported intestinal or immune-mediated disease background (28, 29) who voluntarily followed a 4-week GFD (**Supplementary Table S4** shows the number of samples excluded in the quality control). Circulating miRNA profiles were generated from two plasma samples per individual: one taken during the GFD (4 weeks after start of the GFD) and one taken when eating a regular, gluten-containing diet (either before start of the GFD or after a 2-week wash-out period following the GFD intervention). The study protocol for the GFD cohort was described in detail in Baranska et al. and Bonder et al. (28, 29).

All the protocols of the three studies included in this project were approved by the medical ethics committees of the participating centers and conducted according to the Declaration of Helsinki (15, 27, 29, 30).

### Sample Pre-Processing

Samples were collected for the PreventCD study using BD Vacutainer^®^ SST II Advance (number 367957). Samples were centrifuged for 10 minutes at 3000 RPM after which serum was collected and stored at -80⁰C. For the healthy volunteer GFD cohort, samples were collected using BD Vacutainer^®^ K2E (EDTA) tubes (number 367525). Samples were centrifuged for 10 min at 1300 RPM after which plasma was collected and stored at -80°C.

For the Milano-Bicocca cohort, samples were collected using glass BD Vacutainer^®^ K3EDTA tubes. After collection the tubes were immediately inverted several times to prevent clotting. The samples were maintained at 4°C and processed within 30 min (meaning the time necessary to come back from the hospital). Separation was obtained by centrifugation at 1500 rcf for 15 min in a refrigerated centrifuge and the upper two thirds of the volume was collected to prevent cell contamination. Hemolyzed samples were not collected. Plasma samples were stored at -80°C and shipped to the Netherlands on dry ice.

Previous studies have shown that extracellular microRNA profiles extracted from serum and plasma microRNA are highly correlated (31). However, to avoid bias related to sample type, we did not pool samples from plasma and serum, instead performing separate analyses in the separate cohorts.

### RNA Isolation

Serum or plasma samples (50-250 µl) were centrifuged at 1.000xg for 5 min at 4⁰C to pellet cellular debris. RNA was isolated from the supernatant using the mirVana PARIS kit (Ambion, Carlsbad, CA, USA) according to the manufacturer’s protocol. To increase RNA purity and yield, the acid-chloroform extraction step and RNA elution step were repeated (32). Subsequently, total RNA was precipitated by adding 0.1 volume of 3M Sodium acetate (pH 5.2), 3 volumes 100% molecular-grade ethanol and glycoblue (Ambion). After vortexing, samples were stored at -80⁰C for at least 1 hr. Samples were then centrifuged at maximum speed for 30 min at 4⁰C in an Eppendorf centrifuge. Supernatant was discarded and pellets were washed with 70% molecular-grade ethanol, upon which the samples were centrifuged again for 10 min at 4⁰C. The supernatant was then removed, and the pellet was dried in a vacuum desiccator for 5 min max. The RNA pellet was subsequently re-dissolved in 5 µl RNAse-free water. RNA was isolated from small-intestinal biopsy material with the miRVana kit (Ambion), and small RNA-libraries were generated from 500 ng isolated RNA.

### Small-RNA Library Preparation and Sequencing

Small-RNA libraries were generated as described in the TruSeq Small RNA Sample Prep Kit manual (Illumina, San Diego, CA, USA), performing 15 cycles in the amplification step. In the purification step after cDNA synthesis, glycoblue (Ambion) was used. The cDNA concentration was measured using the LabChip GX (Caliper). Twenty libraries were pooled equimolarly per lane and sequenced on an Illumina HiSeq2500.

### Alignment of miRNA Reads and Quality Control

Raw sequencing reads were trimmed and aligned to the most up-to-date version of the reference database, miRBase 22 (26), using a stand-alone version of sRNAbench (version 1.5 - 6/2018). Default settings were applied, with the exception that the number of mismatches allowed between reference database and reads was set from 1 to 0. We used a cut-off of minimally 100 uniquely aligned miRNAs with >1 read counts and >1,000 read counts aligned to miRNAs in total. Samples that met these criteria were subjected to further Quality Control (QC) steps that are explained in more detail in the **Supplementary Methods**: Quality control of the miRNA profiles.

### Differential Expression Analyses

All statistical analyses were performed in “R” (version 3.5.1). The R-package compareGroups (version 4.0.0) was applied to assess differences in clinical baseline characteristics between cases and controls, including the Shapiro-Wilks test to decide between normally or non-normally distributed variables. Differential expression analysis was performed using the DESeq2 package (version 1.22.2). For further details, including covariates that were taken into account, see **Supplementary Tables S5**–**S7**. P-values for the differential expression analyses and meta-analyses were adjusted for multiple testing using the Benjamini-Hochberg correction for False Discovery Rate (FDR) (33). MiRNAs were considered significantly differentially expressed at an FDR-corrected P-value < 0.1. The R-package Pheatmap (version 1.0.12) was used to create heatmaps to visualize the log2foldchanges of the differential expression analyses. All other figures were generated using the R-package ggplot2 (version 3.1.0). In the figures that display regularized log-normalized miRNA counts, the counts were corrected for batch and age.

### Identification of Circulating miRNAs That Are Early Biomarker Candidates for CeD

To identify circulating miRNAs associated with CeD development, we performed three independent analyses using the PreventCD cohort and the Milano-Bicocca cohort (see **Figure 2**, part 1 *Finding biomarkers for CeD development* and **Table 1**). The results of these three separate analyses were combined to identify which miRNAs showed the most consistent trends over all three analyses. Before this meta-analyses, the Cochrane’s Q test was performed. For all miRNAs that did not show significant heterogeneity (Cochrane’s Q P-value >0.05), a fixed-effects meta-analysis was performed using the inverse-variance method to pool the log2fold changes and their standard errors of different comparisons (meta package, version 4.9-5).

Next, after identifying the miRNAs that show characteristic global trends for CeD development, we zoomed in further to examine more specific trends. To get insight into whether the miRNA levels change depending on how close an individual is to seroconversion, we grouped the pre-diagnostic, TGA negative, samples of the PreventCD patients based on how long before seroconversion they were taken (more than 2 years (>2 years), between 2 and 1 year before diagnosis (2>x>1 years), less than 1 year before diagnosis (<1 year)) and compared these to controls (corrected for sex, age and batch). Samples taken at 4 months of age, i.e. before introduction of gluten, were excluded from this analysis.

A potential source of the circulating miRNAs that are biomarker candidates for CeD is the tissue that is affected in CeD ‒ the small intestine. To investigate whether the circulating miRNAs reflect the intestinal miRNA environment in CeD, we performed a differential expression analysis using the miRNA profile of intestinal biopsies of CeD patients *versus* the profile of control biopsies (patients and control biopsies obtained from Milano-Bicocca cohort participants) and compared these results with the circulating miRNA profile.

### Identification of GFD-Associated miRNAs

To identify miRNAs that change in response to GFD, three different analyses were performed and subsequently combined in a meta-analysis (see comparisons A-C in **Figure 2** – part 2 *Finding miRNAs that change upon gluten-free diet*; **Table 1**). We applied the same statistical methods for the meta-analysis as described above.

### Pathway Analyses

Pathway analyses were performed with the online tool DIANA-miRPath v3.0 database (34). This tool produces a list of genes based on available databases that contain miRNA-gene pairs and performs pathway enrichment analyses using genes that are predicted to be targeted by the set of miRNAs. The standard settings were used, using the KEGG pathways, and only enrichments with FDR <0.05 were considered significant.

## Results

### Cohort Characteristics

We used three cohorts to identify whether miRNAs in circulation could be indicative of CeD (at diagnosis and in timepoints prior to TGA positivity) or change upon initiation of GFD. The clinical parameters of the three cohorts (PreventCD, the Milano-Bicocca cohort and a GFD intervention cohort) are summarized in **Tables S3A**–**1C**, and more detailed participant information for the PreventCD and Milano-Bicocca cohorts is described in the “**Supplementary Methods**: Additional participant characteristics of the PreventCD and Milano-Bicocca cohort”.

**Figure 1:**
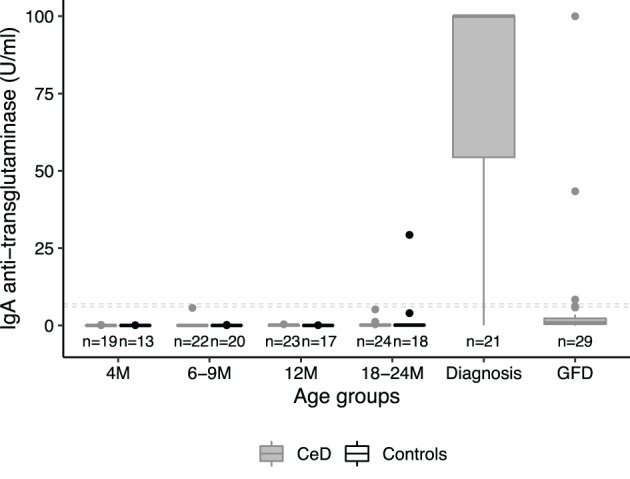
IgA anti-transglutaminase levels peak at diagnosis in the patient group only. IgA anti-transglutaminase levels (TGA) in serum samples of PreventCD participants displayed by age of sampling (CeD=individuals who developed CeD; Ctr=age-matched samples of individuals who did not develop CD; M=Months). For the individuals that developed CeD, we also show serology at diagnosis and after initiating a gluten-free diet (GFD). Samples of individuals in the CeD group that were taken at timepoint of first positive TGA (seroconversion) or at the time of the diagnostic biopsy, were grouped in the diagnosis group (age median: 24, range: 13 - 64 months). One control individual showed positive TGA (29 U/L), but this individual did not have or develop CeD in the follow up (see **Supplemental Methods** for more information). This sample with a positive TGA in the control taken at 3 years of age was grouped with the M18-M24 age group for visualization and analysis purposes. Dashed lines indicate the cutoffs used to assign positivity, depending on the two types of tests used (see *Methods*). Boxplots were generated using the default parameters in the R package ggplot2 (median, second and third quartiles shown by the hinges, individual datapoints are displayed outside the whiskers beyond 1.5 * interquartile range).

In the PreventCD cohort, the duration of follow up did not differ between high-risk participants who did develop CeD during the study and those who did not develop CeD (P=0.38) (see **Table S3A**). The CeD cases carried the DQ2.5/DQ2.5 or the DQ2.5/DQ2.2 HLA haplotype significantly more often compared to participants who did not develop CeD, consistent with what was observed in the full cohort (15). **Figure 1** shows the levels of TGA of the patients in PreventCD divided by age group, at time of diagnosis and after start of the GFD. For the participants that developed CeD, the diagnostic samples were defined throughout the manuscript as the samples at seroconversion (first sample with positive TGA antibodies) or at diagnostic biopsy, and all the negative TGA samples were designated pre-diagnostic timepoint samples. One of the control individuals displayed transiently elevated TGA levels at 3 years of age, but did not develop CeD in follow up (age 9.5). In most patients, TGA levels normalized after start of the GFD (**Figure 1**). More detailed information on the PreventCD participants is provided in the “**Supplementary Methods**: Additional participant characteristics of the PreventCD and Milano-Bicocca cohort”.

Additionally, samples were collected from an independent Milano-Bicocca cross-sectional cohort consisting of pediatric controls, pediatric CeD patients at time of diagnosis and from 10 of these patients 2 years after start of the GFD (see **Supplementary Methods** for more information about the included participants). In the Milano-Bicocca cohort, no differences were observed in age or sex between non-CeD controls and cases at time of CeD diagnosis and after start of the GFD (the results are displayed in **Table S3B**). TGA levels normalized in the majority of patients for whom we also had samples after start of the GFD (**Table S3B**).

The GFD intervention cohort consisted of adults without self-reported intestinal or immune-mediated diseases who voluntarily followed a 4-week GFD. Unfortunately, anti-transglutaminase antibody measurements were not available for this cohort. In the GFD intervention study no differences were observed with regards to the food-related phenotypes measured (mean energy, protein, carb, fat content per day) or with regards to plasma cytokines, when these individuals were on their normal diet *vs* when on GFD (the results are displayed in **Table S3C**) (29).

### Quality Control

After extracting miRNA, library preparation and sequencing, we performed rigorous quality control (QC) to ensure that only high-quality samples were included in our analysis (see “**Supplementary Methods**: Quality control of the miRNA profiles” and **Supplementary Figures S1**–**4** for an overview of the QC workflow, **Supplementary Table S4** for an overview of the samples excluded during the QC). In total, 206 samples of the PreventCD study (82% of the sequenced total; 53 individuals), 52 samples of the Milano-Bicocca cohort (98%; 42 individuals) and 24 samples of the GFD intervention study (100%; 12 individuals) were included for further analysis (an overview of the samples excluded during QC is provided in **Supplementary Table S4**). All 43 miRNA libraries generated from the small-intestinal biopsy RNA available for the Milano-Bicocca cohort passed QC. The reason for the difference in library preparation efficiency between circulating RNA samples and biopsy-derived RNA samples may be that RNA yield from circulation is low and cannot be detected prior to sequencing of the miRNA libraries when starting with the available serum volumes (50-250 µl). The biopsy library preparations were started with a standard 500 ng RNA. High-quality samples were subsequently used for differential expression analysis.

### Circulating miRNAs as Potential Early Biomarkers For CeD Development

**Figure 2:**
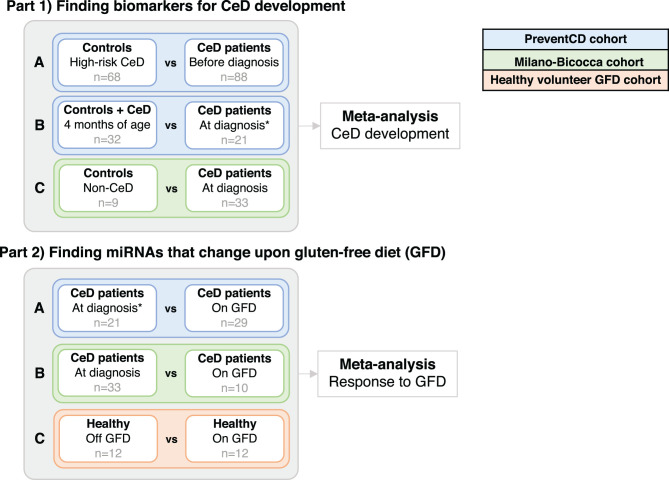
Analyses in the separate cohorts that were performed before combining the results of the differential expression in two meta-analyses. The goals here were to: in part 1) find miRNAs that are potential biomarkers for CeD development and part 2) find miRNAs that change upon the gluten-free diet (GFD). Corresponding sample sizes are shown in grey. *In the PreventCD cohort, the samples “at diagnosis” include samples at seroconversion (first positive IgA anti-transglutaminase (TGA) levels) and samples taken close to the diagnostic biopsy. All samples in the “before diagnosis” groups had negative TGA levels.

To find circulating miRNAs that could function as biomarkers for distinct stages of CeD development, we performed a systematic comparison in three independent cohorts and the results were subsequently summarized in a meta-analysis (see **Figure 2** – part 1 *Finding biomarkers for CeD development* and **Table 1**). The first comparison was performed to identify circulating miRNAs that are predictive markers for CeD development (**Figure 2** part 1, comparison A). Pre-diagnostic samples of children who developed CeD, taken prior to detection of elevated TGA levels, were compared to samples from high-risk controls (the results of this comparison are displayed in **Supplementary Table S5**). The country of sample collection (Netherlands *vs* others) had a limited effect on the differences between CeD and controls: after adding country as a confounder to the statistical analyses, the fold changes between pre-diagnostic samples of CeD and controls were highly correlated to the fold changes without country in the model (R=0.94, P <2.2*10^-16^). Because only one of the control individuals was HLA-DQ2.5 homozygous, we only checked within the patient group whether HLA type had an effect on the miRNA profile (HLA-DQ2.5 homozygous *vs* other HLA). Of the miRNAs significantly different between the pre-diagnostic and control samples, none were significantly different between the HLA groups (FDR>0.3).

Next, in a second comparison, to identify biomarkers at time of diagnosis, we compared the circulating miRNA profile in the PreventCD cohort between diagnostic samples (taken at seroconversion or at diagnostic biopsy) and samples taken at 4 months of age (**Figure 2**, part 1, comparison B; the results of this comparison are displayed in **Supplementary Table S6**). In this comparison, the 4 months samples were used as the baseline because the entire PreventCD cohort is considered free of CeD at this age since gluten has not yet been introduced into their diet. Finally, we used a pediatric case-control cohort (Milano-Bicocca cohort) to find miRNAs that differ between controls and CeD at time of diagnosis (**Figure 2** part 1, comparison C; the results of this comparison are displayed in **Supplementary Table S7**).

**Figure 3:**
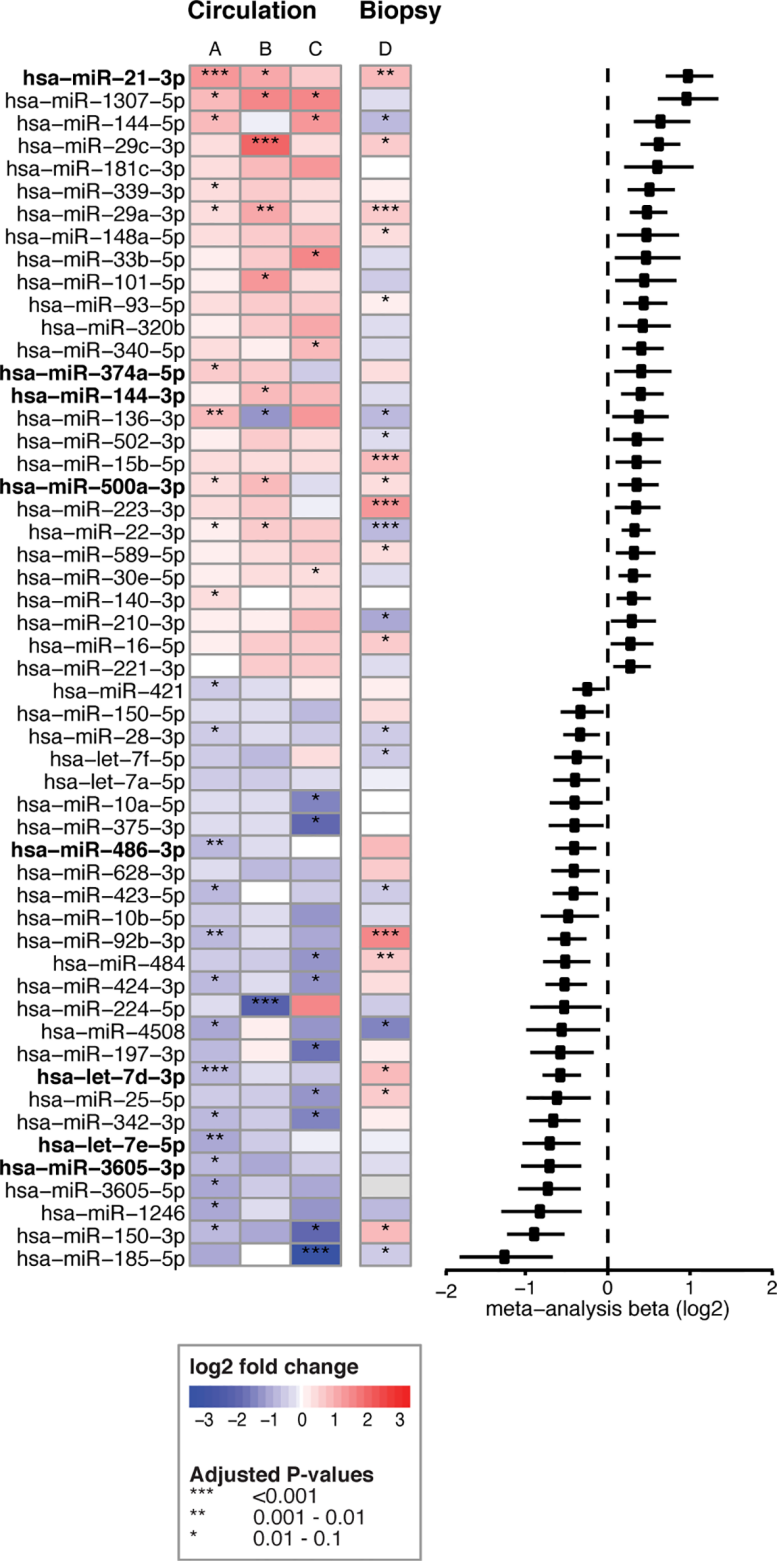
53 circulating miRNA biomarker candidates for CeD development. Log2fold changes are depicted for three separate differential expression (DE) analyses **(A–C)** of 53 microRNAs that were significant in the meta-analysis combining these analyses. **(A)** PreventCD: pre-diagnostic samples of CeD patients (IgA anti-transglutaminase (TGA) negative) *versus* controls. **(B)** PreventCD: CeD at diagnosis (at seroconversion (TGA positivity) or at diagnostic biopsy) *versus* samples at 4 months of age (before gluten consumption). **(C)** Milano-Bicocca: CeD at diagnosis *versus* controls. **(D)** Milano-Bicocca: CeD at diagnosis *versus* controls in intestinal biopsy samples. Right panel shows a forest plot for the meta-analysis (beta and 95% confidence interval). miRNAs that are detectable < 12 months before diagnosis are indicated in bold.

To identify which miRNAs had the most consistent trends over these three comparisons (**Figure 2**, part 1, A-C), we combined the results in a meta-analysis. By considering the effect size (including direction of effect) in the meta-analysis, our results are less dependent on the sample size. This approach identified 53 significant miRNAs that were consistently associated with CeD development (the results of the meta-analysis that combines the results of the three separate comparisons are shown in **Supplementary Table S8**). Of the 53 miRNAs, 26 showed decreased levels in CeD and 27 showed increased levels. The trends for these 53 miRNAs in the three separate analyses (**Figure 2**, part 1, A-C) are displayed in **Figure 3**, including the beta of the meta-analysis that represents the pooled direction across the three comparisons.

To assess the contribution of the Milano-Bicocca cohort, we also performed an additional meta-analysis with only the comparisons performed in the PreventCD cohort (**Figure 2**, part 1, comparisons A-B), yielding 41 significant microRNAs. Of the 53 biomarkers significant in the meta-analysis of comparisons A-C (**Figure 2**, part 1), 29 were also significant in the meta-analysis of comparisons A-B (**Supplementary Table S12**). Moreover, there was a high concordance between the direction of effect between the 53 microRNAs significant in the meta-analyses of arms A-C and that of arms A-B (Pearson’s correlation coefficient of 0.96 (P<2.2*10-16). These results indicate that the addition of the Milano-Bicocca cohort (arm C) adds power to the meta-analysis. Therefore, throughout the manuscript the meta-analysis including arms A-C is used to prioritize the biomarker candidates for CeD development.

**Figure 4:**
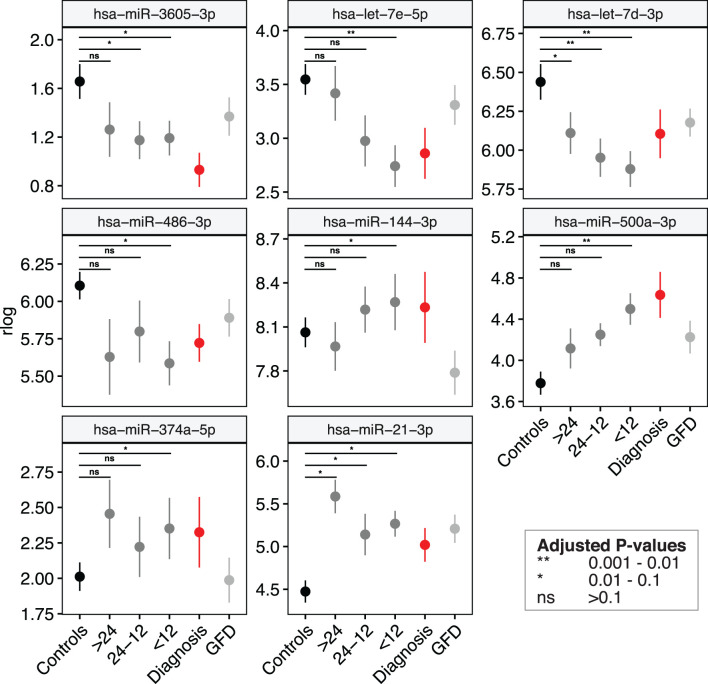
Several miRNA biomarkers for CeD change months to years before detection of CeD serology. The levels of eight out of the 53 microRNAs listed in differ from controls < 12 months before seroconversion (first IgA anti-transglutaminase positivity). Shown are mean values ± standard error of the regularized log-normalized miRNA counts, corrected for batch and age. Black: controls; Dark-grey: pre-diagnostic samples of CeD patients grouped by months till seroconversion (all samples had negative IgA anti-transglutaminase levels); Red: samples at diagnosis (samples at seroconversion or at time of biopsy); grey: CeD patients after start of the GFD.

**Figure 5:**
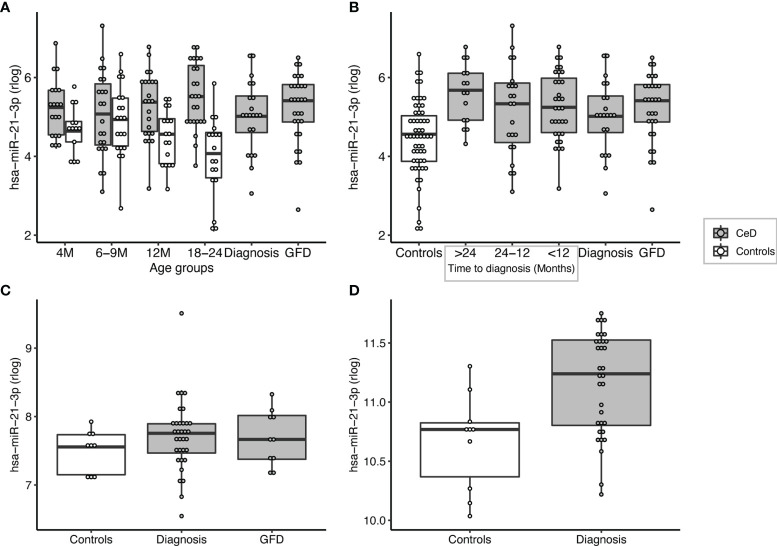
miR-21-3p can be detected at high levels in pre-diagnostic samples of patients but not in age-matched controls and is significantly upregulated in the small intestinal biopsies of CeD patients. **(A)** PreventCD cohort: grouped by age of sampling (M=Months). **(B)** PreventCD cohort: pre-diagnostic (IgA anti-transglutaminase negative) samples of CeD patients are grouped by time till seroconversion: more than 24 months before seroconversion (>24), between 24-12 months before seroconversion (24-12), less than 12 months before seroconversion (<12), or at diagnosis (taken at seroconversion or at time of biopsy) and 6 months after starting GFD. Controls: all samples of the PreventCD controls. **(C)** Circulating miR-21-3p in the Milano-Bicocca cohort (circulation). **(D)** miR-21-3p expression in small-intestinal biopsies in the Milano-Bicocca cohort.

We then zoomed in on specific trends in the prioritized 53 biomarker candidates in the PreventCD cohort, by grouping the pre-diagnostic samples of the CeD patients (all had negative TGA) by how close to seroconversion the samples were taken (<1 year, 1-2 years and >2 years before seroconversion) (the results of these comparisons are displayed in **Supplementary Table S8** and **Supplementary Figures S5, 6**). Eight of the 53 prioritized miRNAs that were identified in the meta-analysis (miR-21-3p, miR-374a-5p, 144-3p, miR-500a-3p, miR-486-3p let-7d-3p, let-7e-5p and miR-3605-3p) are significantly different between the samples taken closest to seroconversion (<1 year) and control samples (the fold changes and adjusted P-values of these comparisons for these eight microRNAs are shown in **Table 2**; the results for all 53 miRNAs are shown in **Supplementary Table S8**). For some of these eight miRNAs, including miR-500a-3p and miR-3605-3p, the levels in pre-diagnostic samples increasingly diverge from controls coming up to seroconversion and diagnosis, and then show a normalizing trend after start of a GFD (see **Table 2** and **Figure 4**). For two of these eight miRNAs, miR-21-3p (shown in **Figure 5**) and let-7d-3p, we detected a significant difference between pre-diagnostic samples and controls more than 2 years before seroconversion and subsequent diagnosis (**Figure 4**).

**Table 2:** Of the 53 circulating miRNA biomarker candidates for CeD development identified in the meta-analysis (**Figure 2**), these eight miRNAs were significantly different in samples taken <12 months before diagnosis.

	Meta-analysis				>24 M *vs* Controls		12-24 M *vs* Controls		<12 M *vs* Controls		Biopsies (CeD *vs* Controls)	
	beta	se	P	P_adj_	log2(FC)	P_adj_	log2(FC)	P_adj_	log2(FC)	P_adj_	log2(FC)	P_adj_
hsa-miR-21-3p	0.99	0.15	1.5E-11	3.9E-09	1.40	4.1E-03	1.25	1.1E-03	1.31	3.5E-04	0.81	4.4E-03
hsa-miR-374a-5p	0.43	0.18	1.6E-02	7.8E-02	0.69	3.3E-01	0.70	3.1E-01	1.10	2.3E-02	0.46	1.6E-01
hsa-miR-144-3p	0.42	0.13	1.4E-03	1.3E-02	-0.15	8.7E-01	0.38	5.3E-01	0.77	3.6E-02	-0.17	7.2E-01
hsa-miR-500a-3p	0.37	0.13	3.3E-03	2.5E-02	0.30	6.4E-01	0.47	3.3E-01	0.96	2.7E-03	0.32	3.0E-02
hsa-miR-486-3p	-0.39	0.13	2.2E-03	1.9E-02	-0.27	6.4E-01	-0.39	3.9E-01	-0.64	7.0E-02	0.74	1.7E-01
hsa-let-7d-3p	-0.56	0.12	3.0E-06	1.3E-04	-0.65	1.0E-01	-0.90	2.8E-03	-0.94	4.8E-04	0.72	8.5E-02
hsa-let-7e-5p	-0.68	0.18	1.4E-04	2.8E-03	-0.27	7.9E-01	-0.89	1.3E-01	-1.53	4.8E-04	-0.01	9.9E-01
hsa-miR-3605-3p	-0.69	0.19	2.3E-04	3.4E-03	-0.82	2.1E-01	-1.08	3.7E-02	-1.09	2.7E-02	-0.20	7.6E-01

Some can even be detected more than 2 years before the first detection of IgA anti-transglutaminase antibodies (seroconversion), >24 M vs Controls. The first set of columns show the results of the meta-analysis. The next three sets of columns show the comparisons in the PreventCD cohort between the samples taken >24 months, 12-24 or <12 months before seroconversion versus control samples [corrected for sex, age and batch and after exclusion of samples taken before introduction of gluten (Month 4)]. The last set of columns shows the comparison between CeD and controls in the small intestinal biopsies (Milano-Bicocca cohort), corrected for age and sex. FC, Fold Change; se, standard error of the beta; Padj, P-value adjusted for multiple testing; Colors, A positive beta or log2(FC) (displayed in green) indicates that the miRNA level is higher in patients who developed CeD; Red, lower in patients who developed CeD; Yellow, Padj<0.1.

To assess the potential influence of age on miRNA levels in controls, we compared the samples taken at 4 and 24 months in controls (**Supplemental Table S11**). This revealed 11 microRNAs that overlapped in the same direction with the comparison M4 *versus* diagnosis (**Figure 2** part 1 comparison B). Only two of these microRNAs (miR-29c and miR-224) were among the 53 biomarker candidates that were prioritized in the final meta-analysis. These results indicate that by combining different comparisons in the meta-analysis, we could filter out most microRNAs for the which the main driver is age-related changes.

Overall, we identified 53 miRNAs that could indicate if a person will develop CeD before the TGA elevation that accompanies intestinal mucosal damage. We hypothesized that the affected tissue in CeD, the small intestine, is a potential source of the 53 CeD-associated circulating miRNAs. Indeed, for the 53 circulating biomarker candidates for CeD, 15 miRNAs are differentially expressed in intestinal biopsies from CeD patients compared to controls, with a concordant direction between circulating and intestinal biopsy‒derived miRNAs. The results of the comparison between CeD and controls in the biopsy material are shown in **Figure 3** and **Supplementary Table S8** for the 53 miRNAs that were identified in meta-analysis A. Two of the eight miRNAs that show an early pre-diagnostic increase in circulation, miR-21-3p (displayed in **Figures 4**, **5**) and miR-500a-3p (displayed in **Figures 4** and **Supplementary S6**), are also significantly increased in CeD biopsies (for the results of the comparison in biopsies see **Table 2**). To check if there was a statistically significant enrichment for upregulated miRNAs in CeD biopsies within the miRNAs that are upregulated in circulation, we used a hypergeometric test considering all miRNAs detected by miRNA-seq in both the biopsies and in plasma samples in the Milano-Bicocca cohort. We found a significant enrichment for these miRNAs (P= 5.1 x 10-6), indicating that there is a higher concordance between the differentially expressed miRNAs in circulation and biopsies beyond what would be expected by chance.

### Circulating Biomarkers in Relation to the Initiation of a Gluten Free Diet

**Figure 6:**
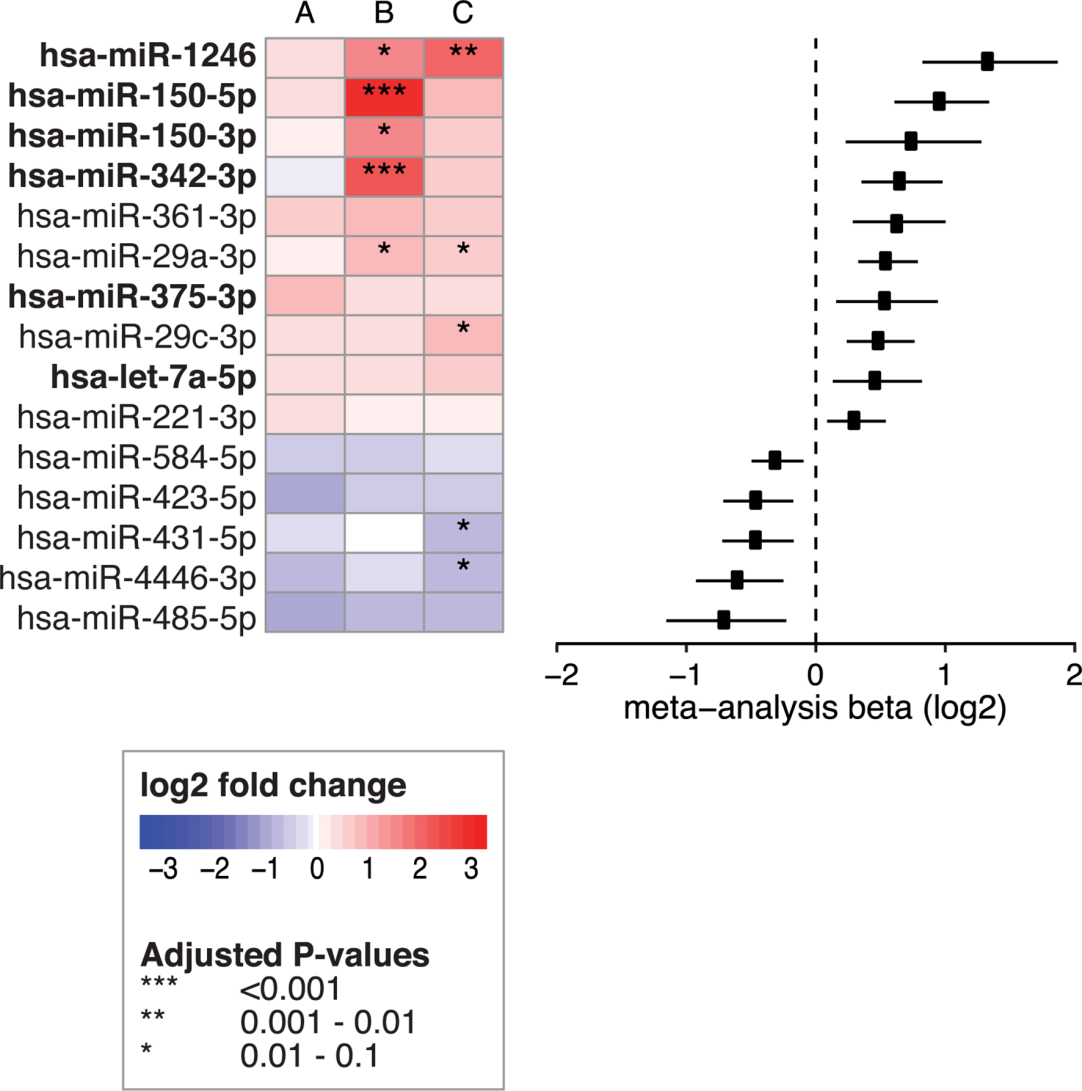
Fifteen circulating miRNAs change after start of the GFD. Left panel shows the 15 circulating miRNAs that were significant in the meta-analysis when combining the following comparisons: **(A)** PreventCD: GFD *vs* CeD at diagnosis (taken at seroconversion or at time of biopsy) **(B)** Milano-Bicocca: GFD *vs* CeD at diagnosis and **(C)** GFD volunteers: GFD *vs* gluten containing diet. Right panel shows forest plot for the meta-analysis (beta and 95% confidence interval). Bold text indicates miRNAs that are also among the 53 CeD biomarker candidates and show a normalizing trend upon GFD.

**Figure 7:**
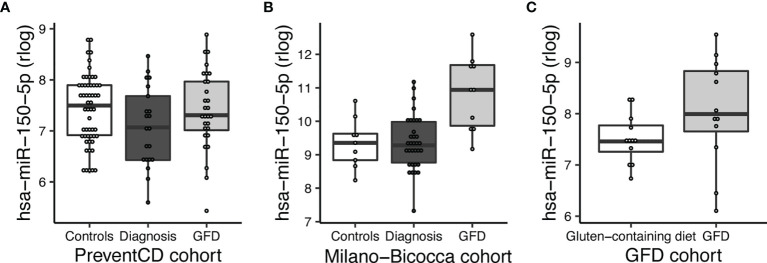
MiR-150-5p is significantly decreased in CeD and reverses after start of a GFD. **(A)** PreventCD: high-risk controls and CeD patients at time of diagnosis (taken at seroconversion or at time of biopsy) and CeD patients after start of a GFD. **(B)** Milano-Bicocca cohort: controls at time of diagnosis (CeD) and at GFD. **(C)** GFD volunteers: on gluten-containing diet or on GFD.

Next, to assess if miRNAs can be used to assess the impact of a GFD, we performed separate comparisons of miRNA profiles of participants on a GFD (**Figure 2** – part 2). These included comparisons in the PreventCD cohort (CeD) (**Figure 2** part 2 comparison A, no miRNAs were significantly differentially expressed), the Milano-Bicocca cohort (CeD) (**Figure 2** part 2 comparison B; significantly differentially expressed miRNAs in this comparison are shown in **Supplementary Table S9**) and the healthy volunteer cohort (**Figure 2** part 2 comparison C; significantly differentially expressed microRNAs in this comparison are shown in **Supplementary Table S10**) and then subsequently combined these results in a meta-analysis. To discern dietary induced microRNA changes from changes due to healing processes in CeD, we have also investigated a cohort of healthy volunteers that were subjected to GFD. In total, 15 circulating miRNAs were significantly associated with the GFD (the results of the meta-analysis are summarized in **Figure 6**). Of the 53 CeD-associated miRNAs, six miRNAs that had decreased levels in circulation at time of diagnosis were significantly increased in response to the GFD: miR-150-5p, miR-150-3p, miR-1246, miR-342-3p, miR-375-3p and let-7a-5p. **Figure 7** shows miR-150-5p, one example of these CeD-associated miRNAs that start to normalize upon GFD. Circulating miR-150-5p increased upon GFD in all 10 individuals for whom we had paired data at diagnosis and after start of the GFD in the Milano-Bicocca cohort. Thus, we were able to identify several miRNAs that can delineate the start of GFD in CeD patients and control individuals.

### Pathway Analyses

We used the DIANA-miRPath v3.0 tool to predict the pathways in which the prioritized circulating miRNAs might play a role. The pathway analysis was performed for the 53 biomarker candidates for CeD development (**Supplementary Figures S7A, B**). The enriched pathways for the miRNAs that were increased in active CeD participants (**Supplementary Figure S7A**) largely overlapped with the pathways found for the miRNAs that decreased upon active CeD (**Supplementary Figure S7B**), as well as the pathways found for the eight miRNAs (**Supplementary Figure S7C**) that increasingly diverge from controls up to diagnosis (shown in **Figure 4**). Top significant pathways include, for example, cell-cycle regulation (hippo signaling pathway, cell-cycle), TGF-beta signaling, fatty-acid metabolism, extracellular matrix interactions and adherence junctions (barrier function). However, because of this overlap, it is difficult to speculate on a functional role for the profiles associated with CeD.

## Discussion

Currently, there are no biomarkers available that can predict the development of CeD before the detection of increased TGA in serum, that is in most cases already accompanied with intestinal mucosal damage. We therefore set out to find novel, non-invasive biomarkers for CeD. For our study, we used three cohorts, including a unique prospective cohort (PreventCD). To our knowledge, our study is the first to apply next generation sequencing to identify miRNAs in circulation in CeD patient samples. By combining the cohorts in a meta-analysis, we identified 53 significant miRNAs that represent potential miRNA biomarker candidates for the development of CeD. Remarkably, eight of these 53 CeD-associated miRNAs could be detected in circulation at an early stage, in some cases more than 2 years before TGA levels were detected above the upper limit of normal. Moreover, we also found six downregulated miRNAs in CeD, including miR-150-3p and miR-150-5p, showed an increased upon a GFD. These miRNA markers are therefore potential markers for CeD, and may be useful for monitoring dietary adherence after start of the GFD. Thus, we have identified a panel of potential miRNA biomarkers that may indicate onset of CeD long before traditional diagnosis of CeD with TGA above the upper limit of normal.

The 53 biomarkers candidates include some miRNAs that have previously been linked to CeD but also some that are being associated with CeD for the first time. For example, Buoli Comani et al. reported that both miR-21-3p and miR-21-5p are highly upregulated in the small intestine of CeD patients and that this elevation was reflected in the circulation (25). This finding was then confirmed by two independent qPCR based studies in which circulating miRNAs were measured (35, 36). Our study, however, is the first to describe that increased levels of miR-21-3p can be detected more than 2 years before the peak in TGA antibodies and the diagnosis of CeD.

Of most interest are the eight miRNAs that were detectable in circulation at a much earlier stage than TGA (in some cases years earlier): miR-21-3p, miR-374a-5p, miR-144-3p, miR-500a-3p, miR-486-3p let-7d-3p, let-7e-5p and miR-3605-3p. For some of these miRNAs, e.g. miR-500a-3p and miR-3605-3p, the difference between pre-diagnostic samples and controls increased depending on how close the samples were taken to the first detection of TGA. In addition, levels of several miRNAs, e.g. miR-500a-3p, normalized after start of a GFD in the PreventCD cohort, although the normalizing effect was not significant. In contrast, miR-21-3p did not (start to) normalize after start of the GFD in the PreventCD cohort. Previously, Bascuñán et al. also reported that miR-21 levels in circulation did not return to normal levels after start of the GFD (37). The observations that miR-21 levels are already elevated more than two years before detection of positive TGA and diagnosis raises the question whether this miRNA is correlated with the development of CeD or rather reflects intrinsic differences between CeD and controls that are independent of the (intestinal) inflammation and intestinal damage. Additionally, the lack of a quick response of these miRNA levels to a GFD might indicate that these miRNAs are not changing because of inflammation/mucosal damage. However, it should be noted that the mucosal healing could take longer than the 6 months after start of the GFD studied in the PreventCD cohort, and adherence to GFD might also influence the response to GFD.

Thus, we observed that that some miRNAs change towards diagnosis (e.g. miR-500a-3p), suggesting that these markers could reflect the pathogenesis of CeD, including immune cell activation, barrier function and mucosal damage. It would be interesting to combine measurements of these miRNAs with other read-outs to detect immune-cell or intestinal function. Other miRNAs, such as miR-21-3p, might represent inherent differences between those individuals who will develop CeD and those who will not, suggesting that this miRNA reflects intrinsic differences between CeD and controls. These intrinsic differences might be linked with factors such as genetic differences and/or immune and intestinal barrier function. Both the biomarkers that reflect the active disease process and the biomarkers that reflect intrinsic risk factors for development of CeD could be valuable in predicting which individuals are at highest risk of developing CeD.

The tissue and cell type of origin for the 53 extracellular circulating microRNAs that we find to be associated with CeD has yet to be uncovered. We did find that 15 of the 53 miRNAs were differentially expressed in active CeD intestinal biopsies, with a concordant direction between circulation and intestinal biopsies. These included the biomarker candidates mentioned above, miR-21-3p and miR-500a, and an increase of miR-21 and miR-500 in CeD biopsies has also been reported by other independent studies (22, 25). Increased miR-21-3p expression in affected gut mucosa has also been described in inflammatory bowel disease (IBD), as has increased expression of the other strand of miR-21 (miR-21-5p) (38, 39). A possible role of miR-21 in intestinal inflammation is also provided by the observation that, in dextran sulphate sodium mouse models, an experimental model for colitis, inflammation is alleviated in miR-21 knock-out mice (40).

This raises the possibility that the 53 miRNAs identified in this study are associated to intestinal inflammation but not specific for CeD. To our knowledge, miR-21-3p in circulation has not been linked to IBD, but the miR-21-5p form is increased in pediatric Crohn’s disease (21). If we also compare the other 53 potential CeD markers with two previous array-based studies in IBD, several microRNAs (miR-16, miR-93 and miR-30e) are elevated in serum of IBD compared to controls (21, 41). However, other microRNAs are elevated in IBD but decreased in the serum of CeD patients, including miR-185, miR-484, miR-25 and members of the let-7 family (21, 41). Therefore, the specificity of this panel of potential biomarkers should be tested, including testing in other intestinal enteropathies and autoimmune diseases.

MiRNAs can function as useful biomarkers but may also have distinct roles in CeD pathophysiology through fine-tuning of gene expression levels. It would be interesting to investigate whether the cell types that play a key role in CeD pathophysiology, e.g. intestinal epithelial cells, gluten-specific T cells or intra-epithelial lymphocytes, selectively secrete or take up miRNAs after the cells are stimulated with compounds that mimic the pathogenic conditions in CeD. Examples of previous efforts to identify the source of CeD-associated miRNAs include those of Bascuñán et al., who showed that miR-21 expression is higher in circulating immune cells (peripheral blood mononuclear cells (PBMC)) isolated from active CeD patients than in PBMC from controls. The levels of miR-21-3p did not increase after stimulation with gliadin and/or interferon-γ. These results indicate that miR-21-3p is expressed by immune cells and, according to reference dataset in peripheral blood, has the highest expression in monocytes, CD4+ and CD8+ T-cells (42).

Predicting miRNA function remains difficult. The functions of individual miRNAs are diverse, as one miRNA can target up to hundreds of genes and one gene can have binding sites for multiple miRNAs (43). This makes it difficult to interpret our pathway analysis results, where we saw overlap between miRNAs increased and decreased in CeD. However, we did find non-immune pathways that have been linked to CeD pathophysiology, such as barrier function (adherence junctions) and fatty acid metabolism, and immune pathways like TGF-beta signaling (44–50). We therefore present the pathway analyses to encourage hypothesis-generation about the potential functions of the circulating miRNA profile associated with CeD but acknowledge that further evidence is needed to confirm that these miRNAs influence these biological pathways.

In summary, we show that circulating miRNAs are promising blood-based biomarker candidates to detect pediatric CeD at an earlier stage than the currently available serological tests. Tests could be designed for these miRNAs that can be more easily implemented in clinics than the next-generation sequencing approach used in this study. However, future independent studies are first needed to confirm whether single or combinations of prioritized miRNAs indeed have value in earlier recognition of CeD in high-risk cohorts. The markers that we found to be associated with the GFD should also be confirmed and compared with other potential markers for gluten intake (such as gluten immunogenic peptides) (51). We did not perform sensitivity/specificity analysis of individual single markers in the current study because testing such statistical prediction models in a cross-validation approach requires a larger sample size, or alternatively needs to be assessed in independent studies. These studies would ideally also test other potential biomarkers for CeD, such as T cell receptor bias, that might also provide specificity and sensitivity, although it is still unclear if these will also be predictive of CeD prior to TGA conversion. It might also be beneficial to measure serum miRNAs in individuals who have positive TGA but no villous atrophy (potential CeD) to see whether the miRNA profile is different between individuals who will develop CeD and those who will not. Moreover, the specificity of the miRNAs to CeD as compared to other immune-mediated diseases, especially those of the gastrointestinal tract, should also be investigated. Finally, future studies should further study factors that could potentially influence circulating miRNA levels, including age (pediatric *vs* controls), genetics (e.g. the role of HLA and regional differences). Nonetheless, our findings hopefully pave the way toward preventative strategies in miRNA-positive individuals in the future, which might minimize the onset of active inflammation, decrease villous atrophy and prevent CeD-associated complications in the future (52).

## Data Availability Statement

The raw data generated for this paper cannot be shared because this possibility was not covered by the Institutional Review Boards agreement when we initiated the study. However, the miRNA count data are available as **Supplementary Material**.

## Ethics Statement

The studies involving human participants were reviewed and approved by the participating centers in the different centers that participated in the three different studies (details were published previously). Written informed consent to participate in this study was provided by the participants’ legal guardian/next of kin.

## Author Contributions

Conceptualization and study design: All authors. Sample collection: DB, CM, MR, EM-O, RS, RA, RT, IK-S, GC, HS, and SK. Sample processing: RCA, RM, and AS. Data analysis and visualization: IT and RCA. Data analysis supervision: VK, YL, IJ, and SW. Writing — original draft preparation: IT, IJ, and SW. Writing — review and editing: RCA, RM, AS, JD, DB, CM, MR, EM-O, RS, RA, RT, IK-S, GC, HS, SK, AZ, VK, YL, MV, RW, MM, and CW. Supervision: SW. All authors contributed to the article and approved the submitted version.

## Funding

IT is supported by a MD/PhD scholarship from the Junior Scientific Masterclass (Graduate School of Medical Sciences, University Medical Center Groningen, and University of Groningen). IJ is supported by a Rosalind Franklin Fellowship from the University of Groningen and a Netherlands Organization for Scientific Research (NWO) VIDI grant (no. 016.171.047). SW and CW were supported by The Netherlands Organ-on-Chip Initiative, an NWO Gravitation project (024.003.001) funded by the Ministry of Education, Culture and Science of the government of The Netherlands; and European Research Council advanced grant (FP7/2007-2013/ERC Advanced Grant Agreement 2012-322698); DB by: 2016-ATE-0312. AZ is supported by the ERC Starting Grant 715772, Netherlands Organization for Scientific Research NWO-VIDI grant 016.178.056, the Netherlands Heart Foundation CVON grant 2018-27, and the NWO Gravitation grant ExposomeNL 024.004.017. YL was supported by an ERC Starting Grant (948207) and the Radboud University Medical Centre Hypatia Grant (2018) for Scientific Research. I.K-S was supported by grants NKFI 120392 from the Hungarian National Research, Development and Innovation Fund and GINOP-2.3.2-15-2016-00015 co-financed by the European Union and the Hungarian State. This study was funded in part by Top Institute Food and Nutrition, Wageningen, The Netherlands, grant number GH001; Stichting Coeliakie Onderzoek Nederland (STICOON); ESPGHAN Networking Grant.

## Conflict of Interest

The authors declare that the research was conducted in the absence of any commercial or financial relationships that could be construed as a potential conflict of interest.

## Publisher’s Note

All claims expressed in this article are solely those of the authors and do not necessarily represent those of their affiliated organizations, or those of the publisher, the editors and the reviewers. Any product that may be evaluated in this article, or claim that may be made by its manufacturer, is not guaranteed or endorsed by the publisher.
