# Supplementary material for: Circulating miRNAs as Potential Biomarkers for Celiac Disease Development
Source: Front Immunol. 2021 Dec 7;12:734763. doi: 10.3389/fimmu.2021.734763 (PMC8688806; doi:10.3389/fimmu.2021.734763)
Supplement: Supplementary file 1 [file DataSheet_1.docx]

**Supplementary Methods****, Tables and Figures**

**Supplementary Methods:** Additional participant characteristics of the PreventCD and Milano-Bicocca cohort**.**

**Supplementary Table S1:** Overview of the control participants in the PreventCD cohort.

**Supplementary Table S2:** Overview of the control participants in the Milano-Bicocca cohort.

**Supplementary table S3.** Clinical characteristics of all three cohorts.

**Supplementary Table S4**. Overview of the microRNA libraries generated from cell-free plasma or serum samples.

**Supplementary Methods:** Quality control of the microRNA profiles

**Supplementary Figure S1**: Steps in the quality control workflow for the microRNA libraries isolated from circulating samples for the PreventCD and the Milano-Bicocca cohort.

**Supplementary Figure S2**: Library size (after alignment) versus the diversity of the library.

**Supplementary Figure S3:** PCA (PC1-4) of the PreventCD cohort

**Supplementary Figure S4:** PCA (PC1–4) of the Milano-Bicocca cohort (n=53).

**Supplementary Table S5.** Results of the differential expression analysis in the circulating microRNA profiles of the PreventCD cohort: pre-diagnostic samples of children who developed CeD versus all control samples, adjusted for sex, batch and age.

**Supplementary Table S6.** Results of the differential expression analysis in the circulating microRNA profiles of the PreventCD cohort: samples taken at diagnosis (taken at seroconversion or at time of diagnostic biopsy) versus samples taken at 4 months of age, adjusted for sex and batch

**Supplementary Table S7.** Results of the differential expression analysis in the circulating microRNA profiles of the Paediatric case-control cohort (Milano-Bicocca cohort): cases at time of diagnosis (n=33) versus controls (n=9), adjusted for batch, age and sex.

**Supplementary Table S8.** Results of the meta-analysis for the 53 circulating miRNA biomarker candidates for CeD development.

**Supplementary Table S9.** Results of the differential expression analysis in the circulating microRNA profiles of the paediatric case-control cohort (Milano-Bicocca cohort).

**Supplementary Table S10.** Results of the differential expression analysis in healthy adult volunteers: GFD versus gluten-containing diet.

**Supplementary Table S11.** Results of the differential expression analysis in PreventCD controls: M4 versus M24.

**Supplementary Table S12.** Results of the meta-analysis CeD development including only the PreventCD comparisons.

**Supplementary Figure S5:** Shows the trend approaching seroconversion and on GFD for the 53 miRNA biomarker candidates.

**Supplementary Figure S6:** miRNA levels for the 53 biomarker candidates (see separate file)

**Supplementary Figure S7:** Pathways associated to the target genes of the 53 circulating biomarker candidates for celiac disease.

**Supplementary Methods**

**Additional participant characteristics of the PreventCD and Milano-Bicocca cohort**

PreventCD cohort

The PreventCD was set up to study whether exposing genetically predisposed infants to dietary gluten at 16 to 24 weeks of age could help to prevent celiac disease (CeD). The results of this trial were previously described in Vriezinga et al *N Engl J Med* 2014^1^. A subset of participants of the full PreventCD cohort were included in the current study.

Among the PreventCD participants who were included in this study, all CeD cases were diagnosed according to the ESPGHAN criteria^2,3^. In all but one patient, the diagnosis included histopathological confirmation of CeD (Marsh 2 or higher). One patient presented with TGA levels greater than 10x the upper limit of normal (100 U/l), clinical symptoms of CeD and HLA-DQ8/DQ7.

Supplementary Table S1 shows an overview of the participants of the PreventCD cohort who did not develop CeD. Of the control group, 9/20 subjects did not develop any CeD-related symptoms or antibody response. Ten subjects showed an early increase in IgA anti-gliadin-antibodies at 6 months of age without any TGA response upon gluten intervention, but they did not develop CeD. This early transient response in anti-gliadin antibodies (>17 U/mL) was shown to not be indicative of CeD^1^. One participant displayed an elevated level of TGA antibodies just above the cut-off value at 3 years of age (29 U/mL), but no abnormal histological findings were found in the biopsies, and this child had not developed CeD at 9.5 years of age and had not again presented with positive TGA antibody levels or with clinical symptoms indicative of CeD at data-freeze in 2017.

Additional characteristics of the PreventCD participants included in this study are displayed in Supplementary Table S3A.

|  | Total controls: n=20 |
| --- | --- |
| No positive TGA or anti-gliadin antibodies | n=9 |
| Transient positive anti-gliadin antibodies, no positive TGA | n=10 |
| Transient positive TGA, no positive anti-gliadin antibodies | n=1 |

**Supplementary Table S1.** Overview of the control participants in the PreventCD cohort

Milano-Bicocca cohort

The controls subjects of the Milano-Bicocca cohort visited the hospital with a range of clinical symptoms (see Supplementary Table S2). For all controls, the small-intestinal biopsies did not show histological abnormalities. Additional characteristics of the Milano-Biocca cohort are displayed in Supplementary table S3B.

|  | Total controls: n=10 |
| --- | --- |
| Classical clinical symptoms CeD* | n=3 |
| Dyspepsia/reflux | n=3 |
| Episode of gastritis | n=1 |
| Previous ileal obstruction | n=1 |
| Ulcerative colitis in remission | n=1 |
| Eosinophilic esophagitis | n=1 |

**Supplementary Table S2.** Overview of the control participants in the Milano-Bicocca cohort. * The three controls that had clinical symptoms suggestive of CeD tested negative for anti-transglutaminase antibodies and anti-endomysial antibodies.

**Supplementary table S3. Clinical characteristics** **of all three cohorts.**

S3A. PreventCD cohort

|  | Controls (n=20) | CeD (n=33) | Comparison  CeD vs Controls |
| --- | --- | --- | --- |
| Center of origin*  LUMC, Leiden, Netherlands UNINA, Naples, Italy Akademia Medyczna Warszawie, Poland HULP, Madrid, Spain HULF – Valencia, Spain Technion, Israel Budapest, Hungary Sant Joan de Reus, Cataluña Munich, Germany | 10 (50.0%) 6 (30.0%) 1 (5.00%) - 1 (5.00%) 1 (5.00%) 1 (5.00%) - - | 14 (42.4%) 4 (12.1%) 1 (3.03%) 1 (3.03%) - - 5 (15.2%) 6 (18.2%) 2 (6.06%) | X^2^;0.8 |
| Age at last follow up at data freeze (years)^#^ | 7.0 (5.5‒8.5) | 7.0 (4.1‒7.5) | MWU; P=0.38 |
| Age at diagnosis (months) | - | 27.9 (23.4‒39.7) | - |
| Marsh classification  3a 3b 3c Without biopsy^+^ | - | 7 (21.2%)  14 (42.4%)  11 (33.3%)  1 (3.0%) | - |
| Sex  Male Female | 6 (30%)  14 (70%) | 10 (30.3%)  23 (69.7%) | X^2^; P=1 |
| HLA risk group^**^  1 2 3 4 5 | 1 (5.0%)  3 (15%)  9 (45%)  2 (10%)  5 (25%) | 15 (45.5%)  4 (12.1%)  11 (33.3%)  1 (3.0%)  2 (6.0%) | X^2^; P=0.005 |
| PreventCD intervention  Gluten Placebo | 14 (54.5%)  6 (45.5%) | 18 (70%)  15 (30%) | X^2^; P=0.41 |
| Size microRNA library | 74 samples  60,520 (24,201–431,506) | 138 samples  70,344 (24,505–238,378) | MWU; P=0.47 |
| Diversity microRNA library | 74 samples  250 (182–435) | 138 samples  261 (187–394) | MWU; P=0.74 |

Variables are displayed as median (interquartile range) or as number (%).

^#^Last follow up is defined as the most recent blood withdrawal at data freeze in 2017.

^+^One patient was diagnosed without performing a duodenal biopsy.

* Chi-squared test (X^2^) was calculated on proportion Netherlands vs other centers in the CeD vs Control group.

**X^2^ was calculated on proportion HLA risk group 1 vs other groups in the CeD vs Control group. Group 1: DQ2.5/DQ2.5 or DQ2.5/DQ2.2; group 2: DQ2.2/DQ7; group 3: DQ2.5/DQ7, DQ2.5/DQ8 or DQ2.5/other HLA haplotype; group 4: DQ2.2/DQ2.2, DQ2.2/DQ8 or DQ8/DQ8; group 5: DQ2.2/other, DQ8/DQ7 or DQ8/other.

MWU: Mann–Whitney U test.

S3B. Paediatric Milano-Bicocca cohort

|  | Controls  N=10 | CeD (DG)  n=33 | CeD (GFD)  n=10 | Comparison across groups | Post-hoc  DG vs Control | Post-hoc  DG vs GFD |
| --- | --- | --- | --- | --- | --- | --- |
| Age (years) | 10.5 (8.3–12.0) | 8.0 (4.0–13.0) | 8.0 (6.0–11.8) | Kruskal-Wallis; 0.573624 | - | - |
| Sex  Male  Female | 6 (60%)  4 (40%) | 12 (36.6%)  21 (63.4%) | 4 (40%) 6 (60%) | X^2^; 0.485646 | - | - |
| Marsh classification  3a 3b  3a or 3b 3c Unknown | - | 4 (12%)  6 (18%)  4 (12%)  17 (52%)  2 (6%) | - | - | - | - |
| Antibodies (IgA)*  TGA positive**  EMA positive  EMA & TGA positive | 0/4 (0%)  0/4 (0%)  0/4 (0%) | 32/32 (100%)  32/32 (100%)  32/32 (100%) | 3/10 (30%)  4/10 (40%)  1/10 (10%) | X^2^;  <0.000001  <0.000001 | <0.000001  <0.000001 | 0.000006  0.000052 |
|  |  |  |  |  |  |  |
| Size miRNA library | 245720  (128372–301646) | 760940  (537379–1683059) | 452235  (288660–738702) | Kruskal-Wallis;  0.000065 | MWU;  P=000104 | MWU;  P=0.041240 |
| Diversity miRNA library | 309  (269–334) | 469  (419–621) | 406  (353–491) | Kruskal-Wallis;  0.000097 | MWU;  P=0.000171 | MWU; P=0.054072 |

Variables are displayed as median (interquartile range), or as number (% in which this variable was measured). DG: diagnosis.

*Displayed as positive cases/cases in which the cases were measured.

** Antibody positivity was determined using the cutoff values corresponding to the TGA assay that was used.

S3C. Adult volunteer GFD cohort

|  | Gluten-containing diet (n=12) | Gluten-free diet (GFD) (n=12) | P-value  Gluten containing diet vs GFD |
| --- | --- | --- | --- |
| Age (years) at sample collection | 44.2 (31.0‒52.6) |  | - |
| Sex  Male  Female | 4 (33.3%)  8 (66.7%) |  | - |
| Average daily intake nutrients  Energy (kcal)  Protein (g)  Carbohydrates (g)  Fat (g) | 1456 (1394‒2064)  76.9 (58.6– 82.9)  178 (131–229)  66.2 (49.7–84.1) | 1709 (1493–2087)  73.0 (63.6–81.4)  194 (178–236)  68.4 (51.6–83.8) | 0.627207  0.964784  0.309880  0.964784 |
| Cytokines (plasma)  Citrulline (mol/L)  IL-1 Beta (g/L)  IL-6 (g/L)  IL-8 (g/L) IL-10 (g/L) IL-12P70 (g/L)  TNF-Alpha (g/L) | 41.4 (34.7–44.7)  1.01 (0.21–1.79)  0.38 (0.38–1.60)  5.41 (3.16–8.28)  0.88 (0.72–1.01)  0.95 (0.95–1.67)  0.16 (0.16–0.56) | 46.0 (35.6–57.0)  1.23 (1.12–1.68)  0.38 (0.38–0.38)  4.96 (3.12–7.77)  0.88 (0.81–0.94)  1.53 (0.95–2.11)  1.53 (0.16–5.13) | 0.418923  0.638263  0.102470  0.953940  0.861017  0.474760  0.487674 |
| Size miRNA library (24 samples) | 387254  (111790–591781) | 243636  (181543–467096) | MWU: P=0.77 |
| Diversity miRNA library (24 samples) | 414  (276–470) | 377  (328–428) | MWU: P=0.77 |

Variables are displayed as median (interquartile range) or as number (% of cohort).

**Supplementary Table S4**. Overview of the microRNA libraries generated from cell-free plasma or serum samples.

|  |  | Differential expression analysis | CeD  Number of samples | Controls  Number of samples |
| --- | --- | --- | --- | --- |
| **PreventCD** | Age: 4 months | Included | 19* | 13 |
| Total after QC: |  | Excluded | 3 | 5 |
| 206 samples | Age: 6–9 months | Included | 22* | 20 |
|  |  | Excluded | 12 | 2 |
|  | Age: 12 months | Included | 23* | 17 |
|  |  | Excluded | 8 | 3 |
|  | Age: 18–24 months | Included | 24* | 18 |
|  |  | Excluded |  | 2 |
|  | Diagnosis | Included | 21 |  |
|  |  | Excluded | 8 |  |
|  | GFD | Included | 29 |  |
|  |  | Excluded | 1 |  |
| **Milano-Bicocca cohort** | Controls | Included |  | 9 |
| Total after QC: |  | Excluded |  | 1 |
| 52 samples | Diagnosis |  | 33 |  |
|  | GFD |  | 10 |  |
| **GFD-cohort** | Gluten containing diet |  |  | 12 |
| After QC: 24 | GFD |  |  | 12 |

Total number of samples included in the final differential expression analyses are shown in black. The number of microRNA libraries excluded during quality control are shown in red. In the PreventCD cohort, the samples in the “diagnosis” group were taken at seroconversion (first sample with positive TGA) or at diagnostic biopsy (age median: 24, range: 13–64 months). The samples included in the “diagnosis” group of the PreventCD cohort were taken on average 1.71 months after seroconversion.

* Includes only the pre-diagnostic samples of children who developed CeD during the study, before seroconversion (positive TGA).

**Supplementary Methods:** Quality control of the microRNA profiles

**Supplementary Figure S1**: Steps in the quality control workflow for the microRNA libraries isolated from circulating samples for the PreventCD and the Milano-Bicocca cohort.

To ensure the inclusion of only high-quality microRNA libraries in the final differential expression analyses, the quality of the microRNA-libraries was assessed according to the quality control (QC) workflow presented in Figure S1.

In the PreventCD cohort, 20 out of 250 miRNA libraries were removed from further analyses because of low library diversity (less than 100 different aligned miRNAs) and/or less than 1,000 total library size (see supplementary Figures S1A QC step 1, and supplementary Figure S2). To further ensure technical quality of the samples, Principal Component Analyses (PCA) (using the R-package “stats”, version 3.5.1) were performed using the regularized log transformed microRNA counts. An additional 11 microRNA libraries were excluded from further analyses (QC step 2) as outliers (outside the 99% confidence interval calculated per batch) based on the PCA (PC1-4), after exclusion of samples in step 1 (Figure S3A-B). No common clinical parameters could be identified to explain why these samples were outliers. At least two of the 11 microRNA outlier libraries showed a technical bias. The total library size of these two libraries consisted of over 80% one microRNA (miR-10b-3p). In the samples that passed final QC, the maximum percentage that one microRNA contributed was 54.6%.

The regularized log counts were recalculated for the remaining samples (n=219), and the PCA was repeated (QC step 3). We recalculated that PCA after QC step 1 and 2 because PCA depends on the samples included in the analysis and we wanted to remove outliers that persist after exclusion of samples in step 1 and 2. A cluster of 13 samples still clustered away from the other samples, and these were excluded from further analyses. Upon further inspection of the QC parameters, 12 of these 13 samples had a lower sequencing quality and significantly fewer sequencing reads (before alignment to the reference database) compared to the other 66 samples sequenced on the same day (median sequences 77,255 (IQR: 1,671–19,851) versus median 5,251,116 (IQR: 2,001,781–8,280,411), p-value < 0.001, MWU). Finally, for the PreventCD cohort, 206 samples and 285 miRNAs were included in the DE analysis (Figure S3C–D).

All the circulating miRNA libraries in the paediatric Milano-Bicocca cohort and the GFD cohort had sufficient library diversity and size (see Supplementary Figure S1B). One circulating microRNA library of the Milano-Bicocca cohort was removed from the analyses because it clustered away from the other samples sequenced in the same batch in the PCA (Supplementary Figure S4).

All 43 miRNA libraries generated from the small-intestinal biopsy RNA available for the Milano-Bicocca cohort passed QC: all libraries had a library size >6,000,000 and diversity >460 and no outliers were present in the PCA outside the 99% confidence interval within the controls or CeD.

microRNA reads considered in the final analyses:

In previous control libraries prepared from samples containing water only, two miRNAs (hsa-miR-486-5p, and hsa-miR-3168) were detected at unusually high levels (data not shown). These miRNAs were therefore considered to be potential false positives and were excluded from our analyses. Only miRNAs for which more than 5 counts could be detected in the samples that passed QC, per cohort, were considered in further analyses.

**Supplementary Figure S2**: Library size (after alignment) versus the diversity of the library (number of different microRNAs that have >1 count after alignment) in: **A)** all samples (n=327) of the GFD cohort (n=24), Milano-Bicocca cohort (n=33) and PreventCD cohort (n=250). All samples of the GFD and Milano-Bicocca cohort pass the quality control filtering threshold of QC step 1 (represented by the horizontal and vertical bars). **B–D):** PreventCD cohort (n=250). **B)** shows which samples are excluded in the different QC steps that are presented in Figure S1. High quality libraries could be generated for samples that originated from different centers **(C)** and with different volumes of serum **(D)**.

Center of origin: 1: LUMC, Leiden, Netherlands; 2: UNINA, Naples, Italy; 3: Akademia Medyczna Warszawie, Poland; 4: HULP, Madrid, Spain; 5: HULF – Valencia, Spain; 6: Technion, Israel 8: Budapest, Hungary; 9: Sant Joan de Reus, Cataluña; 10: Munich, Germany

**Supplementary Figure S3: A–B)** PCA (PC1–4) of the PreventCD cohort after exclusion of samples that did not pass QC step 1 (n=230). Samples in red were marked as outliers by this PCA as they were outside the 99% CI calculated per batch (QC step 2). When repeating this analysis after excluding the samples of step 1 and 2, the samples in orange were marked as step 3 outliers. **C–D)** PCA (PC1–4) of the PreventCD cohort with only the final samples included, no samples were outside the 99% CI calculated per batch. As one clear technical covariate (batch, represented by differences in input material) was present in PC1, the sample batch was used as a technical covariate to correct for this effect in all subsequent differential expression analyses.

**Supplementary Figure S4:** PCA (PC1–4) of the Milano-Bicocca cohort (n=53). The ellipse indicates the 99% confidence level calculated per batch (represented by sequencing date). One sample was considered to be an outlier (see black arrow) and removed from further analyses (QC step 2). As one clear technical covariate (batch, represented by sequencing) was present in PC1/2, the sample batch was used as a technical covariate to correct for this batch effect in all subsequent differential expression analyses.

|  | **baseMean** | **log2FoldChange*** | **lfcSE** | **Adjusted P-value** |
| --- | --- | --- | --- | --- |
| hsa-miR-21-3p | 73 | 1.17 | 0.20 | 6.58E-07 |
| hsa-miR-136-3p | 8 | 0.82 | 0.22 | 3.50E-03 |
| hsa-miR-345-5p | 41 | 0.76 | 0.17 | 3.35E-04 |
| hsa-miR-1307-5p | 83 | 0.76 | 0.24 | 1.73E-02 |
| hsa-miR-144-5p | 59 | 0.73 | 0.24 | 2.22E-02 |
| hsa-miR-134-5p | 12 | 0.68 | 0.27 | 7.79E-02 |
| hsa-miR-141-3p | 12 | 0.65 | 0.19 | 1.01E-02 |
| hsa-miR-29b-3p | 5 | 0.61 | 0.24 | 7.72E-02 |
| hsa-miR-374a-5p | 6 | 0.58 | 0.23 | 7.88E-02 |
| hsa-miR-654-3p | 27 | 0.54 | 0.21 | 7.72E-02 |
| hsa-miR-629-5p | 8 | 0.53 | 0.23 | 9.89E-02 |
| hsa-miR-339-3p | 7 | 0.50 | 0.19 | 6.93E-02 |
| hsa-miR-410-3p | 45 | 0.48 | 0.20 | 9.69E-02 |
| hsa-miR-500a-3p | 22 | 0.43 | 0.17 | 7.66E-02 |
| hsa-miR-140-3p | 215 | 0.40 | 0.14 | 3.96E-02 |
| hsa-miR-29a-3p | 65 | 0.37 | 0.14 | 7.66E-02 |
| hsa-miR-22-3p | 4888 | 0.26 | 0.11 | 9.69E-02 |
| hsa-miR-28-3p | 213 | -0.33 | 0.14 | 8.92E-02 |
| hsa-miR-421 | 17 | -0.38 | 0.13 | 3.98E-02 |
| hsa-miR-15a-5p | 1016 | -0.42 | 0.18 | 9.89E-02 |
| hsa-miR-652-3p | 67 | -0.45 | 0.18 | 7.72E-02 |
| hsa-miR-424-3p | 42 | -0.52 | 0.16 | 1.70E-02 |
| hsa-miR-92b-3p | 220 | -0.56 | 0.15 | 3.50E-03 |
| hsa-miR-423-5p | 8228 | -0.57 | 0.19 | 2.22E-02 |
| hsa-miR-342-3p | 50 | -0.58 | 0.20 | 3.98E-02 |
| hsa-let-7b-5p | 155 | -0.60 | 0.16 | 3.50E-03 |
| hsa-miR-92a-3p | 11221 | -0.64 | 0.13 | 1.59E-04 |
| hsa-miR-486-3p | 67 | -0.66 | 0.17 | 3.05E-03 |
| hsa-let-7d-3p | 80 | -0.68 | 0.15 | 4.14E-04 |
| hsa-miR-3605-3p | 3 | -0.69 | 0.23 | 2.86E-02 |
| hsa-miR-150-3p | 17 | -0.70 | 0.22 | 2.14E-02 |
| hsa-let-7c-5p | 43 | -0.75 | 0.20 | 4.18E-03 |
| hsa-miR-4508 | 4 | -0.79 | 0.30 | 6.67E-02 |
| hsa-miR-3605-5p | 5 | -0.82 | 0.26 | 2.03E-02 |
| hsa-miR-4433b-5p | 9 | -0.82 | 0.34 | 9.69E-02 |
| hsa-miR-1246 | 518 | -0.89 | 0.34 | 7.10E-02 |
| hsa-miR-342-5p | 19 | -0.90 | 0.20 | 4.18E-04 |
| hsa-let-7e-5p | 13 | -0.91 | 0.23 | 1.88E-03 |
| hsa-miR-4492 | 5 | -1.23 | 0.40 | 2.44E-02 |
| hsa-miR-4454 | 11 | -1.31 | 0.32 | 1.76E-03 |
| hsa-miR-133a-3p | 18 | -1.58 | 0.37 | 8.92E-04 |
| hsa-miR-122-5p | 45 | -1.95 | 0.51 | 3.48E-03 |

**Supplementary Table S5:** Results of the differential expression analysis in the circulating microRNA profiles of the PreventCD cohort: pre-diagnostic samples of children who developed CeD (anti-transglutaminase antibodies were negative) versus all control samples, adjusted for sex, batch and age. This comparison corresponds to comparison 1A in Figure 2.

*Positive log2FoldChanges indicate that the microRNA levels are higher in pre-diagnostic CeD than in controls.

|  | **baseMean** | **log2FoldChange*** | **lfcSE**** | **Adjusted P-value** |
| --- | --- | --- | --- | --- |
| hsa-miR-29b-3p | 8 | 2.05 | 0.45 | 4.09E-04 |
| hsa-miR-29c-3p | 25 | 1.78 | 0.29 | 8.42E-08 |
| hsa-miR-1307-5p | 111 | 1.34 | 0.39 | 1.36E-02 |
| hsa-miR-101-5p | 2 | 1.20 | 0.41 | 4.34E-02 |
| hsa-miR-106b-5p | 34 | 1.18 | 0.27 | 6.07E-04 |
| hsa-miR-17-3p | 6 | 1.09 | 0.30 | 6.17E-03 |
| hsa-miR-29a-3p | 83 | 1.01 | 0.25 | 2.60E-03 |
| hsa-miR-21-3p | 84 | 1.01 | 0.33 | 3.42E-02 |
| hsa-miR-451a | 4436 | 0.93 | 0.25 | 6.17E-03 |
| hsa-miR-3158-3p | 3 | 0.92 | 0.38 | 9.81E-02 |
| hsa-let-7i-3p | 6 | 0.89 | 0.33 | 6.90E-02 |
| hsa-miR-141-3p | 16 | 0.87 | 0.30 | 4.65E-02 |
| hsa-miR-142-3p | 34 | 0.85 | 0.29 | 4.34E-02 |
| hsa-miR-144-3p | 394 | 0.81 | 0.28 | 4.34E-02 |
| hsa-miR-215-5p | 108 | 0.81 | 0.31 | 7.61E-02 |
| hsa-miR-378c | 35 | 0.76 | 0.29 | 7.61E-02 |
| hsa-miR-500a-3p | 28 | 0.74 | 0.27 | 6.12E-02 |
| hsa-miR-652-3p | 78 | 0.74 | 0.29 | 9.08E-02 |
| hsa-miR-182-5p | 451 | 0.73 | 0.29 | 9.58E-02 |
| hsa-miR-25-3p | 1663 | 0.72 | 0.24 | 4.34E-02 |
| hsa-miR-15a-5p | 1112 | 0.71 | 0.29 | 9.81E-02 |
| hsa-miR-363-3p | 212 | 0.70 | 0.23 | 3.23E-02 |
| hsa-miR-101-3p | 585 | 0.62 | 0.23 | 6.12E-02 |
| hsa-miR-425-5p | 118 | 0.54 | 0.22 | 9.58E-02 |
| hsa-miR-22-3p | 5978 | 0.52 | 0.18 | 5.04E-02 |
| hsa-miR-27b-3p | 1135 | -0.53 | 0.17 | 3.06E-02 |
| hsa-miR-199a-3p | 141 | -0.53 | 0.21 | 9.58E-02 |
| hsa-miR-199b-3p | 141 | -0.53 | 0.21 | 9.58E-02 |
| hsa-miR-127-3p | 134 | -0.80 | 0.29 | 6.33E-02 |
| hsa-miR-410-3p | 44 | -0.86 | 0.31 | 5.72E-02 |
| hsa-miR-432-5p | 9 | -1.01 | 0.37 | 6.50E-02 |
| hsa-miR-136-3p | 8 | -1.03 | 0.35 | 4.34E-02 |
| hsa-miR-654-3p | 28 | -1.19 | 0.35 | 1.65E-02 |
| hsa-miR-125b-2-3p | 7 | -1.25 | 0.39 | 2.70E-02 |
| hsa-miR-323a-3p | 1 | -1.40 | 0.54 | 7.61E-02 |
| hsa-miR-369-3p | 3 | -1.45 | 0.44 | 1.75E-02 |
| hsa-miR-134-5p | 12 | -1.52 | 0.41 | 5.82E-03 |
| hsa-miR-411-5p | 10 | -1.55 | 0.37 | 1.33E-03 |
| hsa-miR-433-3p | 1 | -1.58 | 0.61 | 7.72E-02 |
| hsa-miR-224-5p | 4 | -1.92 | 0.44 | 6.07E-04 |
| hsa-miR-483-5p | 15 | -5.74 | 0.58 | 9.18E-21 |
| hsa-miR-483-3p | 4 | -6.07 | 0.73 | 9.05E-15 |

**Supplementary Table S6** Results of the differential expression analysis in the circulating microRNA profiles of the PreventCD cohort: samples taken at diagnosis (taken at seroconversion or at time of diagnostic biopsy) versus samples taken at 4 months of age, adjusted for sex and batch. This comparison corresponds to comparison 1B in Figure 2.

*Positive log2FoldChanges indicate that the microRNA levels are higher at time of diagnosis compared to the samples taken at 4 months of age that are considered free of active CeD. **lfcSE: standard error of the log2 fold change.

|  | **baseMean** | **log2FoldChange*** | **lfcSE** | **Adjusted P-value** | **Levels going up at GFD Cases/total (%)** |
| --- | --- | --- | --- | --- | --- |
| hsa-miR-98-3p | 4 | 2.29 | 0.81 | 4.57E-02 | 60% |
| hsa-miR-548bc | 5 | 2.19 | 0.74 | 3.68E-02 | 40% |
| hsa-miR-374a-3p | 10 | 2.09 | 0.66 | 2.59E-02 | 60% |
| hsa-miR-1273h-3p | 5 | 2.09 | 0.76 | 5.16E-02 | 80% |
| hsa-miR-493-5p | 4 | 1.90 | 0.78 | 9.60E-02 | 70% |
| hsa-miR-28-5p | 45 | 1.80 | 0.44 | 3.38E-03 | 70% |
| hsa-miR-30b-5p | 118 | 1.72 | 0.43 | 3.38E-03 | 70% |
| hsa-miR-381-3p | 14 | 1.69 | 0.71 | 9.97E-02 | 70% |
| hsa-miR-625-5p | 15 | 1.52 | 0.58 | 5.99E-02 | 50% |
| hsa-miR-148b-5p | 17 | 1.50 | 0.43 | 1.06E-02 | 40% |
| hsa-miR-33b-5p | 36 | 1.40 | 0.47 | 3.68E-02 | 30% |
| hsa-miR-1307-5p | 195 | 1.39 | 0.51 | 5.47E-02 | 30% |
| hsa-miR-26a-5p | 8028 | 1.32 | 0.36 | 9.78E-03 | 70% |
| hsa-miR-548o-3p | 12 | 1.31 | 0.40 | 2.16E-02 | 50% |
| hsa-miR-410-3p | 35 | 1.26 | 0.50 | 7.69E-02 | 60% |
| hsa-miR-194-5p | 98 | 1.24 | 0.46 | 5.50E-02 | 60% |
| hsa-miR-144-5p | 772 | 1.14 | 0.36 | 2.57E-02 | 30% |
| hsa-miR-424-5p | 16 | 1.14 | 0.39 | 3.68E-02 | 80% |
| hsa-miR-21-5p | 3495 | 1.13 | 0.26 | 1.21E-03 | 70% |
| hsa-miR-10399-3p | 8 | 1.11 | 0.46 | 9.71E-02 | 60% |
| hsa-miR-152-3p | 37 | 1.07 | 0.39 | 5.50E-02 | 60% |
| hsa-miR-15a-5p | 6655 | 1.00 | 0.35 | 4.42E-02 | 40% |
| hsa-miR-744-5p | 179 | 0.98 | 0.38 | 7.04E-02 | 50% |
| hsa-miR-17-3p | 82 | 0.93 | 0.37 | 8.29E-02 | 30% |
| hsa-miR-181c-5p | 119 | 0.92 | 0.26 | 1.06E-02 | 80% |
| hsa-miR-142-3p | 349 | 0.92 | 0.31 | 3.68E-02 | 70% |
| hsa-let-7d-5p | 637 | 0.92 | 0.33 | 4.79E-02 | 70% |
| hsa-miR-340-5p | 145 | 0.91 | 0.29 | 3.03E-02 | 70% |
| hsa-miR-301a-3p | 113 | 0.86 | 0.31 | 4.57E-02 | 70% |
| hsa-miR-26b-5p | 1752 | 0.85 | 0.32 | 5.73E-02 | 80% |
| hsa-miR-181b-5p | 244 | 0.66 | 0.25 | 5.67E-02 | 80% |
| hsa-miR-181a-2-3p | 39 | 0.59 | 0.24 | 9.71E-02 | 90% |
| hsa-miR-126-3p | 655 | 0.56 | 0.19 | 4.15E-02 | 40% |
| hsa-miR-30e-5p | 5383 | 0.49 | 0.18 | 5.50E-02 | 40% |
| hsa-miR-345-5p | 297 | -0.54 | 0.19 | 4.42E-02 | 30% |
| hsa-miR-186-5p | 7715 | -0.63 | 0.22 | 4.42E-02 | 20% |
| hsa-miR-532-5p | 980 | -0.69 | 0.26 | 5.50E-02 | 20% |
| hsa-miR-941 | 309 | -0.73 | 0.31 | 9.97E-02 | 70% |
| hsa-miR-7706 | 66 | -0.92 | 0.35 | 5.99E-02 | 10% |
| hsa-miR-424-3p | 183 | -0.93 | 0.35 | 5.50E-02 | 20% |
| hsa-miR-484 | 1735 | -0.98 | 0.34 | 4.42E-02 | 40% |
| hsa-miR-25-5p | 21 | -1.05 | 0.35 | 3.68E-02 | 40% |
| hsa-miR-191-5p | 16197 | -1.07 | 0.40 | 5.73E-02 | 60% |
| hsa-miR-629-5p | 34 | -1.18 | 0.38 | 2.89E-02 | 30% |
| hsa-miR-342-3p | 273 | -1.20 | 0.41 | 3.68E-02 | 100% |
| hsa-miR-10a-5p | 4428 | -1.26 | 0.42 | 3.68E-02 | 60% |
| hsa-miR-5001-3p | 8 | -1.33 | 0.39 | 1.46E-02 | 40% |
| hsa-miR-197-3p | 59 | -1.34 | 0.47 | 4.37E-02 | 100% |
| hsa-miR-24-3p | 253 | -1.41 | 0.40 | 1.06E-02 | 70% |
| hsa-miR-183-5p | 657 | -1.44 | 0.38 | 5.32E-03 | 30% |
| hsa-miR-3613-5p | 17 | -1.48 | 0.43 | 1.26E-02 | 60% |
| hsa-miR-99b-5p | 369 | -1.55 | 0.45 | 1.26E-02 | 50% |
| hsa-miR-375-3p | 535 | -1.65 | 0.59 | 4.91E-02 | 70% |
| hsa-miR-150-3p | 28 | -1.68 | 0.47 | 1.06E-02 | 90% |
| hsa-miR-96-5p | 178 | -1.71 | 0.44 | 3.99E-03 | 20% |
| hsa-miR-1294 | 36 | -2.05 | 0.52 | 3.66E-03 | 30% |
| hsa-miR-501-3p | 147 | -2.20 | 0.37 | 4.40E-07 | 30% |
| hsa-miR-185-5p | 78 | -3.55 | 0.60 | 4.40E-07 | 40% |
| hsa-miR-122-5p | 281 | -4.36 | 0.87 | 7.11E-05 | 40% |

**Supplementary Table S7** Results of the differential expression analysis in the circulating microRNA profiles of the Paediatric case-control cohort (Milano-Bicocca cohort): cases at time of diagnosis (n=33) versus controls (n=9), adjusted for batch, age and sex. This comparison corresponds to comparison 1C in Figure 2.

*Positive log2FoldChanges indicate that the microRNA levels are higher at time of diagnosis compared to the controls.

| **Meta-analysis** | | | | **>24 M vs Controls** | | **12-24 M vs Controls** | | **<12 M vs Controls** | | **Biopsies (CeD vs Controls)** | |  |
| --- | --- | --- | --- | --- | --- | --- | --- | --- | --- | --- | --- | --- |
| **beta** | **se** | **P** | **P_adj_** | **log2(FC)** | **P_adj_** | **log2(FC)** | **P_adj_** | **log2(FC)** | **P_adj_** | **log2(FC)** | **P_adj_** |  |
| hsa-miR-21-3p | 0.99 | 0.15 | 1.5E-11 | 3.9E-09 | 1.40 | 4.1E-03 | 1.25 | 1.1E-03 | 1.31 | 3.5E-04 | 0.81 | 4.4E-03 |
| hsa-miR-1307-5p | 0.98 | 0.19 | 1.8E-07 | 1.3E-05 | 0.86 | 2.0E-01 | 0.91 | 1.4E-01 | 0.47 | 5.1E-01 | -0.11 | 8.5E-01 |
| hsa-miR-144-5p | 0.66 | 0.18 | 1.7E-04 | 3.1E-03 | 0.95 | 1.3E-01 | 0.82 | 2.0E-01 | 0.40 | 5.9E-01 | -0.59 | 8.8E-02 |
| hsa-miR-29c-3p | 0.64 | 0.12 | 1.9E-07 | 1.3E-05 | 0.20 | 8.3E-01 | 0.52 | 3.4E-01 | 0.70 | 1.2E-01 | 0.54 | 5.5E-02 |
| hsa-miR-181c-3p | 0.62 | 0.22 | 3.9E-03 | 2.8E-02 | 0.67 | 4.4E-01 | 0.22 | 8.1E-01 | 0.12 | 9.2E-01 | 0.07 | 8.2E-01 |
| hsa-miR-339-3p | 0.53 | 0.15 | 2.9E-04 | 3.9E-03 | 0.60 | 2.9E-01 | 0.65 | 2.0E-01 | 0.31 | 6.1E-01 | 0.10 | 6.4E-01 |
| hsa-miR-29a-3p | 0.50 | 0.12 | 2.0E-05 | 6.6E-04 | 0.23 | 6.8E-01 | 0.20 | 7.1E-01 | 0.51 | 1.9E-01 | 0.65 | 1.9E-04 |
| hsa-miR-148a-5p | 0.49 | 0.19 | 1.1E-02 | 5.7E-02 | 0.85 | 2.2E-01 | 0.51 | 5.4E-01 | 0.15 | 8.6E-01 | 0.37 | 6.7E-02 |
| hsa-miR-33b-5p | 0.48 | 0.20 | 1.7E-02 | 7.9E-02 | 0.06 | 9.6E-01 | 0.22 | 8.1E-01 | 0.11 | 9.3E-01 | -0.16 | 7.9E-01 |
| hsa-miR-101-5p | 0.46 | 0.19 | 1.5E-02 | 7.3E-02 | 0.10 | 9.5E-01 | 0.75 | 2.8E-01 | 0.59 | 4.1E-01 | -0.33 | 4.6E-01 |
| hsa-miR-93-5p | 0.46 | 0.14 | 8.3E-04 | 8.9E-03 | 0.08 | 9.5E-01 | 0.55 | 3.1E-01 | 0.67 | 1.2E-01 | 0.24 | 6.5E-02 |
| hsa-miR-320b | 0.45 | 0.16 | 6.4E-03 | 4.1E-02 | 0.63 | 3.6E-01 | 0.24 | 7.6E-01 | 0.32 | 6.7E-01 | -0.21 | 5.4E-01 |
| hsa-miR-340-5p | 0.43 | 0.13 | 8.6E-04 | 8.9E-03 | 0.84 | 3.2E-02 | 0.23 | 6.9E-01 | 0.21 | 6.9E-01 | -0.10 | 7.8E-01 |
| hsa-miR-374a-5p | 0.43 | 0.18 | 1.6E-02 | 7.8E-02 | 0.69 | 3.3E-01 | 0.70 | 3.1E-01 | 1.10 | 2.3E-02 | 0.46 | 1.6E-01 |
| hsa-miR-144-3p | 0.42 | 0.13 | 1.4E-03 | 1.3E-02 | -0.15 | 8.7E-01 | 0.38 | 5.3E-01 | 0.77 | 3.6E-02 | -0.17 | 7.2E-01 |
| hsa-miR-136-3p | 0.40 | 0.17 | 2.3E-02 | 9.4E-02 | 1.08 | 5.0E-02 | 0.63 | 3.1E-01 | 0.45 | 5.1E-01 | -0.63 | 1.8E-02 |
| hsa-miR-502-3p | 0.37 | 0.16 | 1.6E-02 | 7.8E-02 | 0.01 | 9.9E-01 | 0.60 | 3.3E-01 | 0.55 | 3.8E-01 | -0.25 | 8.2E-02 |
| hsa-miR-15b-5p | 0.37 | 0.14 | 8.2E-03 | 4.9E-02 | 0.16 | 8.7E-01 | 0.13 | 8.1E-01 | 0.39 | 4.6E-01 | 0.87 | 1.3E-05 |
| hsa-miR-500a-3p | 0.37 | 0.13 | 3.3E-03 | 2.5E-02 | 0.30 | 6.4E-01 | 0.47 | 3.3E-01 | 0.96 | 2.7E-03 | 0.32 | 3.0E-02 |
| hsa-miR-223-3p | 0.36 | 0.14 | 1.0E-02 | 5.7E-02 | 0.45 | 4.8E-01 | 0.08 | 8.8E-01 | 0.30 | 6.2E-01 | 1.21 | 8.4E-06 |
| hsa-miR-22-3p | 0.34 | 0.09 | 1.0E-04 | 2.1E-03 | 0.25 | 5.3E-01 | 0.30 | 3.5E-01 | 0.30 | 3.3E-01 | -0.71 | 1.0E-08 |
| hsa-miR-589-5p | 0.34 | 0.12 | 5.7E-03 | 3.7E-02 | 0.43 | 3.9E-01 | 0.41 | 3.5E-01 | 0.14 | 8.2E-01 | 0.50 | 2.7E-02 |
| hsa-miR-30e-5p | 0.33 | 0.10 | 1.0E-03 | 1.0E-02 | 0.08 | 9.5E-01 | 0.41 | 3.1E-01 | 0.32 | 4.2E-01 | -0.16 | 5.1E-01 |
| hsa-miR-140-3p | 0.31 | 0.11 | 2.9E-03 | 2.3E-02 | 0.41 | 3.5E-01 | 0.51 | 2.0E-01 | 0.27 | 5.3E-01 | 0.09 | 6.8E-01 |
| hsa-miR-210-3p | 0.31 | 0.14 | 2.4E-02 | 9.7E-02 | -0.17 | 8.6E-01 | 0.55 | 3.1E-01 | 0.19 | 7.7E-01 | -0.84 | 1.3E-02 |
| hsa-miR-16-5p | 0.30 | 0.13 | 2.5E-02 | 9.7E-02 | -0.27 | 6.6E-01 | 0.24 | 7.1E-01 | 0.24 | 6.7E-01 | 0.61 | 1.6E-02 |
| hsa-miR-221-3p | 0.29 | 0.12 | 1.1E-02 | 5.7E-02 | -0.26 | 6.4E-01 | 0.12 | 8.1E-01 | -0.10 | 8.6E-01 | -0.22 | 1.2E-01 |
| hsa-miR-421 | -0.23 | 0.10 | 2.2E-02 | 9.4E-02 | -0.15 | 7.9E-01 | -0.20 | 6.7E-01 | -0.46 | 1.4E-01 | 0.15 | 5.4E-01 |
| hsa-miR-150-5p | -0.31 | 0.13 | 2.0E-02 | 8.6E-02 | -0.16 | 8.6E-01 | -0.30 | 6.2E-01 | 0.08 | 9.2E-01 | 0.36 | 3.0E-01 |
| hsa-miR-28-3p | -0.32 | 0.11 | 5.3E-03 | 3.6E-02 | 0.16 | 8.3E-01 | -0.44 | 3.3E-01 | -0.30 | 5.1E-01 | -0.32 | 7.6E-02 |
| hsa-let-7f-5p | -0.36 | 0.15 | 1.7E-02 | 8.0E-02 | 0.07 | 9.6E-01 | -0.39 | 5.9E-01 | -0.58 | 2.6E-01 | -0.43 | 3.0E-02 |
| hsa-let-7a-5p | -0.38 | 0.14 | 9.3E-03 | 5.3E-02 | -0.09 | 9.5E-01 | -0.24 | 7.1E-01 | -0.60 | 1.9E-01 | -0.06 | 8.9E-01 |
| hsa-miR-10a-5p | -0.38 | 0.16 | 1.9E-02 | 8.6E-02 | -0.10 | 9.5E-01 | -0.88 | 9.2E-02 | -0.23 | 7.6E-01 | 0.02 | 9.5E-01 |
| hsa-miR-375-3p | -0.38 | 0.17 | 2.5E-02 | 9.7E-02 | -0.34 | 6.4E-01 | -0.62 | 3.1E-01 | -0.27 | 6.9E-01 | 0.06 | 8.8E-01 |
| hsa-miR-486-3p | -0.39 | 0.13 | 2.2E-03 | 1.9E-02 | -0.27 | 6.4E-01 | -0.39 | 3.9E-01 | -0.64 | 7.0E-02 | 0.74 | 1.7E-01 |
| hsa-miR-628-3p | -0.39 | 0.15 | 8.6E-03 | 5.0E-02 | -0.13 | 9.4E-01 | -0.14 | 8.2E-01 | -0.36 | 5.9E-01 | 0.60 | 1.4E-01 |
| hsa-miR-423-5p | -0.39 | 0.14 | 4.9E-03 | 3.4E-02 | -0.58 | 3.1E-01 | -0.71 | 1.4E-01 | -0.49 | 3.6E-01 | -0.45 | 9.6E-02 |
| hsa-miR-10b-5p | -0.46 | 0.18 | 1.1E-02 | 5.7E-02 | 0.01 | 1.0 | -0.99 | 8.0E-02 | -0.42 | 5.4E-01 | -0.12 | 6.5E-01 |
| hsa-miR-92b-3p | -0.49 | 0.12 | 4.1E-05 | 1.1E-03 | -0.69 | 5.7E-02 | -1.17 | 1.4E-05 | -0.51 | 1.2E-01 | 1.37 | 4.4E-04 |
| hsa-miR-484 | -0.50 | 0.15 | 6.8E-04 | 8.3E-03 | -0.95 | 3.4E-02 | -0.53 | 3.1E-01 | -0.24 | 6.9E-01 | 0.63 | 3.1E-03 |
| hsa-miR-424-3p | -0.50 | 0.13 | 8.3E-05 | 1.9E-03 | -1.08 | 1.0E-02 | -0.39 | 4.1E-01 | -0.23 | 6.7E-01 | 0.40 | 3.2E-01 |
| hsa-miR-224-5p | -0.51 | 0.22 | 2.1E-02 | 9.0E-02 | 0.24 | 8.6E-01 | -0.69 | 3.9E-01 | -0.65 | 4.2E-01 | -0.32 | 2.2E-01 |
| hsa-miR-4508 | -0.54 | 0.23 | 1.9E-02 | 8.5E-02 | -1.10 | 1.9E-01 | -0.23 | 8.1E-01 | -0.09 | 9.4E-01 | -1.15 | 5.6E-02 |
| hsa-miR-197-3p | -0.56 | 0.20 | 4.6E-03 | 3.3E-02 | -0.85 | 3.1E-01 | -0.91 | 2.4E-01 | -0.09 | 9.4E-01 | 0.28 | 5.4E-01 |
| hsa-let-7d-3p | -0.56 | 0.12 | 3.0E-06 | 1.3E-04 | -0.65 | 1.0E-01 | -0.90 | 2.8E-03 | -0.94 | 4.8E-04 | 0.72 | 8.5E-02 |
| hsa-miR-25-5p | -0.60 | 0.20 | 2.8E-03 | 2.3E-02 | 0.18 | NA | -0.76 | 4.1E-01 | -0.09 | 9.5E-01 | 0.69 | 7.4E-02 |
| hsa-miR-342-3p | -0.64 | 0.16 | 5.0E-05 | 1.2E-03 | -0.71 | 2.2E-01 | -1.07 | 1.1E-02 | -0.43 | 4.7E-01 | 0.27 | 3.0E-01 |
| hsa-let-7e-5p | -0.68 | 0.18 | 1.4E-04 | 2.8E-03 | -0.27 | 7.9E-01 | -0.89 | 1.3E-01 | -1.53 | 4.8E-04 | -0.01 | 9.9E-01 |
| hsa-miR-3605-3p | -0.69 | 0.19 | 2.3E-04 | 3.4E-03 | -0.82 | 2.1E-01 | -1.08 | 3.7E-02 | -1.09 | 2.7E-02 | -0.20 | 7.6E-01 |
| hsa-miR-3605-5p | -0.71 | 0.19 | 2.5E-04 | 3.6E-03 | -0.89 | 2.9E-01 | -1.17 | 7.2E-02 | -0.72 | 3.6E-01 | NA | NA |
| hsa-miR-1246 | -0.80 | 0.25 | 1.3E-03 | 1.2E-02 | -1.48 | 1.0E-01 | -2.76 | 1.5E-05 | -1.05 | 2.2E-01 | -0.63 | 1.9E-01 |
| hsa-miR-150-3p | -0.87 | 0.18 | 9.7E-07 | 5.2E-05 | -0.94 | 1.3E-01 | -0.73 | 2.7E-01 | -0.17 | 8.6E-01 | 0.76 | 6.0E-02 |
| hsa-miR-185-5p | -1.23 | 0.29 | 1.8E-05 | 6.6E-04 | -2.18 | 3.2E-02 | -1.20 | 3.1E-01 | -0.47 | 7.0E-01 | -0.49 | 6.8E-02 |

**Supplementary Table S8.** Results of the meta-analysis for the 53 circulating miRNA biomarker candidates for CeD development. The first set of columns show the results of the meta-analysis (see also Figure 3). The next three sets of columns show the comparisons in the PreventCD cohort between the samples taken >24 months, 12-24 or <12 months before seroconversion versus control samples (corrected for sex, age and batch and after exclusion of samples taken before introduction of gluten (Month 4)). The last set of columns shows the comparison between CeD and controls in the small intestinal biopsies (Milano-Bicocca cohort). FC: Fold Change. se: standard error of the beta. P_adj_: P-value adjusted for multiple testing. A positive beta or log2(FC) indicates that the miRNA level is higher in patients who developed CeD.

**Supplementary Figure S5:** Shows the trend approaching seroconversion (>24, 12–24 and <12 months before seroconversion) and on GFD for the 53 miRNA biomarker candidates. Fold-changes and p-values of the comparisons are displayed in Supplementary Table 8. Shown are the mean values ± the standard error of the regularized log-normalized miRNA counts, corrected for batch and age.

**Supplementary Figure S6 (see separate file):** miRNA levels for the 53 biomarker candidates for **A**) the PreventCD cohort grouped by age of sampling (M=Months), **B**) PreventCD (circulation): Controls: all samples of the PreventCD controls. CeD more than 24 months before seroconversion (>24), between 24–12 months before seroconversion (24–12), less than 12 months before seroconversion (<12), or at diagnosis (at seroconversion or at time of diagnostic biopsy) and 6 months after starting GFD, **C**) Milano-Bicocca cohort (circulation) and **D**) expression in small-intestinal biopsies of the Milano-Bicocca cohort.

|  | **baseMean** | **log2FoldChange*** | **lfcSE** | **Adjusted P-value** | **Levels going up at GFD Cases/total (%)** |
| --- | --- | --- | --- | --- | --- |
| hsa-miR-122-5p | 281 | 3.34 | 0.83 | 3.33E-03 | 40% |
| hsa-miR-150-5p | 1878 | 2.66 | 0.41 | 2.33E-08 | 100% |
| hsa-miR-342-3p | 273 | 2.13 | 0.38 | 4.42E-06 | 100% |
| hsa-miR-197-3p | 59 | 2.06 | 0.43 | 1.88E-04 | 100% |
| hsa-miR-873-5p | 4 | 1.91 | 0.57 | 2.49E-02 | 80% |
| hsa-miR-155-5p | 48 | 1.60 | 0.39 | 2.34E-03 | 90% |
| hsa-miR-150-3p | 28 | 1.49 | 0.44 | 2.29E-02 | 90% |
| hsa-miR-146b-5p | 1062 | 1.47 | 0.46 | 4.53E-02 | 70% |
| hsa-miR-146b-3p | 18 | 1.40 | 0.39 | 1.50E-02 | 90% |
| hsa-miR-1246 | 73 | 1.38 | 0.45 | 5.52E-02 | 80% |
| hsa-miR-342-5p | 75 | 1.36 | 0.30 | 5.17E-04 | 70% |
| hsa-miR-223-3p | 1042 | 1.10 | 0.38 | 8.80E-02 | 90% |
| hsa-miR-29a-3p | 316 | 0.90 | 0.30 | 6.09E-02 | 70% |
| hsa-miR-204-5p | 8 | -2.00 | 0.59 | 2.29E-02 | 30% |

**Supplementary Table S9.** Results of the differential expression analysis in the circulating microRNA profiles of the paediatric case-control cohort (Milano-Bicocca cohort). Samples taken 2 years after start of the GFD (n=10) are compared to samples taken at diagnosis (n=33) in the paediatric case-control, correcting for batch. This comparison corresponds to comparison 2B in Figure 2. The ID of the individual was also included in the DESeq2 model in order to perform paired analyses.

*Positive log2FoldChanges indicate that the microRNA levels are higher after start of the GFD compared to samples taken at time of diagnosis.

|  | **baseMean** | **log2FoldChange*** | **lfcSE** | **Adjusted P-value** |
| --- | --- | --- | --- | --- |
| hsa-miR-122-5p | 43 | 2.38 | 0.75 | 5.50E-02 |
| hsa-miR-1246 | 22 | 1.94 | 0.43 | 2.36E-03 |
| hsa-miR-29c-3p | 36 | 0.81 | 0.23 | 3.27E-02 |
| hsa-miR-29a-3p | 95 | 0.58 | 0.15 | 1.21E-02 |
| hsa-miR-431-5p | 47 | -0.52 | 0.15 | 3.65E-02 |
| hsa-miR-4446-3p | 64 | -0.65 | 0.20 | 3.65E-02 |
| hsa-miR-671-3p | 55 | -0.68 | 0.17 | 1.21E-02 |
| hsa-miR-203a-3p | 6 | -1.53 | 0.43 | 2.99E-02 |

**Supplementary Table S10.** Results of the differential expression analysis in healthy adult volunteers: GFD versus gluten-containing diet. ID of the individual was also included in the DESeq2 model in order to perform paired analyses. This comparison corresponds to comparison 1C in Figure 2.

*Positive log2FoldChanges indicate that the microRNA levels are higher in the samples taken at GFD compared to the samples taken at regular gluten-containing diet.

|  | **baseMean** | **log2FoldChange*** | **lfcSE** | **Adjusted P-value** |
| --- | --- | --- | --- | --- |
| hsa-let-7i-3p ^+^ | 7 | 1,53 | 0,58 | 9,97E-02 |
| hsa-miR-15a-5p ^+^ | 1578 | 1,53 | 0,46 | 4,32E-02 |
| hsa-miR-4732-3p | 38 | 1,52 | 0,42 | 1,71E-02 |
| hsa-miR-29c-3p ^+^ | 21 | 1,48 | 0,46 | 4,89E-02 |
| hsa-miR-451a ^+^ | 4525 | 1,08 | 0,39 | 9,33E-02 |
| hsa-miR-151a-3p | 480 | -0,91 | 0,34 | 9,97E-02 |
| hsa-miR-181b-5p | 132 | -1,13 | 0,42 | 9,97E-02 |
| hsa-miR-181a-5p | 2025 | -1,19 | 0,39 | 5,88E-02 |
| hsa-miR-191-5p | 1451 | -1,26 | 0,47 | 9,97E-02 |
| hsa-miR-500a-3p | 21 | -1,44 | 0,54 | 9,97E-02 |
| hsa-miR-127-3p ^+^ | 189 | -1,46 | 0,51 | 8,67E-02 |
| hsa-miR-455-5p | 13 | -1,51 | 0,57 | 9,97E-02 |
| hsa-miR-146b-5p | 162 | -1,59 | 0,57 | 9,33E-02 |
| hsa-miR-409-3p | 54 | -1,70 | 0,54 | 5,88E-02 |
| hsa-miR-125b-2-3p ^+^ | 7 | -1,74 | 0,66 | 9,97E-02 |
| hsa-miR-411-5p ^+^ | 11 | -1,90 | 0,67 | 9,33E-02 |
| hsa-miR-432-5p ^+^ | 13 | -1,94 | 0,50 | 7,36E-03 |
| hsa-miR-873-5p | 5 | -1,96 | 0,67 | 8,25E-02 |
| hsa-miR-224-5p ^+^ | 7 | -2,10 | 0,68 | 5,88E-02 |
| hsa-miR-873-3p | 5 | -3,36 | 0,69 | 8,51E-05 |
| hsa-miR-483-3p ^+^ | 10 | -4,73 | 0,87 | 7,77E-06 |
| hsa-miR-483-5p ^+^ | 34 | -5,80 | 0,82 | 5,15E-10 |

**Supplementary Table S11.** Results of the differential expression analysis in PreventCD controls: M4 versus M24.

*Positive log2FoldChanges indicate that the microRNA levels are higher in the samples taken at M24 compared to the samples taken at M4.

+ Are also significant (with the same direction of effect) in the comparison M4 versus Diagnosis in the PreventCD cohort (see Supplementary Table S6).

| **Meta-analysis** | | | |  |
| --- | --- | --- | --- | --- |
| **beta** | **se** | **P** | **P_adj_** |  |
| **hsa-miR-21-3p** | 1.13 | 0.17 | 2.6E-11 | 6.9E-09 |
| **hsa-miR-1307-5p** | 0.91 | 0.20 | 5.8E-06 | 3.9E-04 |
| hsa-miR-141-3p | 0.71 | 0.16 | 1.0E-05 | 4.4E-04 |
| **hsa-miR-29c-3p** | 0.70 | 0.14 | 9.3E-07 | 1.2E-04 |
| hsa-miR-200a-3p | 0.67 | 0.27 | 1.4E-02 | 8.4E-02 |
| hsa-miR-345-5p | 0.65 | 0.14 | 5.2E-06 | 3.9E-04 |
| **hsa-miR-374a-5p** | 0.60 | 0.20 | 2.2E-03 | 2.1E-02 |
| **hsa-miR-339-3p** | 0.54 | 0.16 | 9.1E-04 | 1.1E-02 |
| **hsa-miR-500a-3p** | 0.51 | 0.14 | 2.8E-04 | 4.8E-03 |
| **hsa-miR-144-5p** | 0.51 | 0.20 | 1.2E-02 | 7.5E-02 |
| **hsa-miR-93-5p** | 0.43 | 0.15 | 5.7E-03 | 4.8E-02 |
| **hsa-miR-223-3p** | 0.41 | 0.15 | 6.0E-03 | 4.9E-02 |
| **hsa-miR-15b-5p** | 0.38 | 0.15 | 1.1E-02 | 7.3E-02 |
| **hsa-miR-144-3p** | 0.35 | 0.14 | 1.3E-02 | 8.0E-02 |
| **hsa-miR-22-3p** | 0.33 | 0.09 | 4.4E-04 | 5.9E-03 |
| **hsa-miR-140-3p** | 0.31 | 0.12 | 8.7E-03 | 6.1E-02 |
| hsa-miR-199a-3p | -0.30 | 0.11 | 7.9E-03 | 5.9E-02 |
| hsa-miR-199b-3p | -0.30 | 0.11 | 7.7E-03 | 5.9E-02 |
| **hsa-miR-28-3p** | -0.30 | 0.12 | 1.0E-02 | 6.8E-02 |
| **hsa-miR-421** | -0.35 | 0.11 | 1.9E-03 | 1.9E-02 |
| **hsa-miR-484** | -0.39 | 0.16 | 1.6E-02 | 9.1E-02 |
| **hsa-miR-423-5p** | -0.40 | 0.16 | 1.1E-02 | 7.3E-02 |
| **hsa-let-7a-5p** | -0.42 | 0.16 | 8.2E-03 | 6.0E-02 |
| **hsa-miR-424-3p** | -0.44 | 0.14 | 1.6E-03 | 1.7E-02 |
| **hsa-miR-92b-3p** | -0.46 | 0.13 | 3.5E-04 | 5.2E-03 |
| **hsa-let-7f-5p** | -0.47 | 0.16 | 3.9E-03 | 3.5E-02 |
| **hsa-miR-486-3p** | -0.51 | 0.14 | 4.3E-04 | 5.9E-03 |
| **hsa-miR-342-3p** | -0.54 | 0.17 | 1.6E-03 | 1.7E-02 |
| hsa-let-7b-3p | -0.57 | 0.20 | 4.7E-03 | 4.0E-02 |
| **hsa-let-7d-3p** | -0.58 | 0.13 | 8.1E-06 | 4.4E-04 |
| hsa-let-7c-5p | -0.62 | 0.17 | 3.2E-04 | 5.0E-03 |
| **hsa-miR-3605-5p** | -0.69 | 0.22 | 2.0E-03 | 2.0E-02 |
| hsa-miR-4286 | -0.72 | 0.30 | 1.8E-02 | 9.8E-02 |
| **hsa-miR-1246** | -0.72 | 0.29 | 1.3E-02 | 8.0E-02 |
| **hsa-miR-3605-3p** | -0.73 | 0.20 | 2.8E-04 | 4.8E-03 |
| **hsa-miR-150-3p** | -0.74 | 0.19 | 1.2E-04 | 2.3E-03 |
| **hsa-let-7e-5p** | -0.80 | 0.20 | 4.3E-05 | 1.1E-03 |
| hsa-miR-98-3p | -0.81 | 0.27 | 3.1E-03 | 2.8E-02 |
| hsa-miR-4454 | -1.21 | 0.28 | 2.0E-05 | 6.7E-04 |
| hsa-miR-133a-3p | -1.30 | 0.32 | 5.7E-05 | 1.3E-03 |
| hsa-miR-122-5p | -1.49 | 0.44 | 7.6E-04 | 9.7E-03 |

**Supplementary Table S11.** Results of the meta-analysis CeD development including only the PreventCD comparisons (see *Figure 2, part 1, A-B*). This meta-analysis was performed to assess the impact of the addition of the Milano-Biccoca comparison (*Figure 2, part 1, C)*. microRNAs indicated in bold are also significant in the meta-analysis that was used to prioritize biomarker candidates (Supplementary Table S8). se: standard error of the beta. P_adj_: P-value adjusted for multiple testing. A positive beta indicates that the miRNA level is higher in patients who developed CeD.

**Supplementary Figure S7:** Pathways associated to the target genes of the 53 circulating biomarker candidates for celiac disease (CeD). Pathways associated to the target genes of: **A**) the 26 miRNAs that are decreased in circulation of CeD patients, **B**) the 27 miRNAs that are decreased in circulation of CeD patients and **C)** the 8 prioritized miRNAs that are significantly different between the samples taken closest to diagnosis (<1 year before seroconversion) and control samples.
