# Supplementary material for: Circulating miRNAs as Potential Biomarkers for Celiac Disease Development
Source: Front Immunol. 2021 Dec 7;12:734763. doi: 10.3389/fimmu.2021.734763 (PMC8688806; doi:10.3389/fimmu.2021.734763)
Supplement: Supplementary file 2 [file DataSheet_2.pdf]

hsa−miR−3605−3p

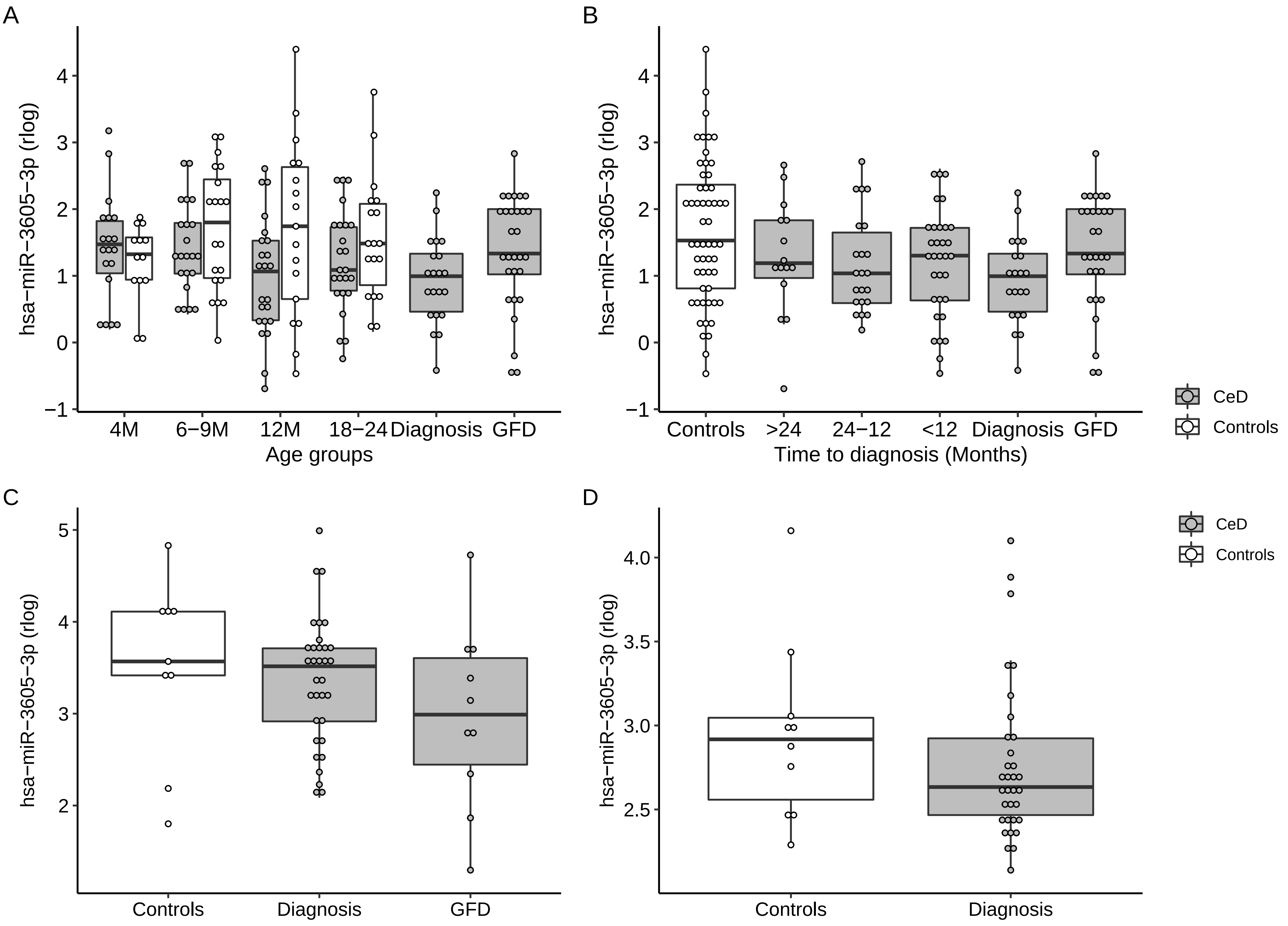

hsa−let−7e−5p

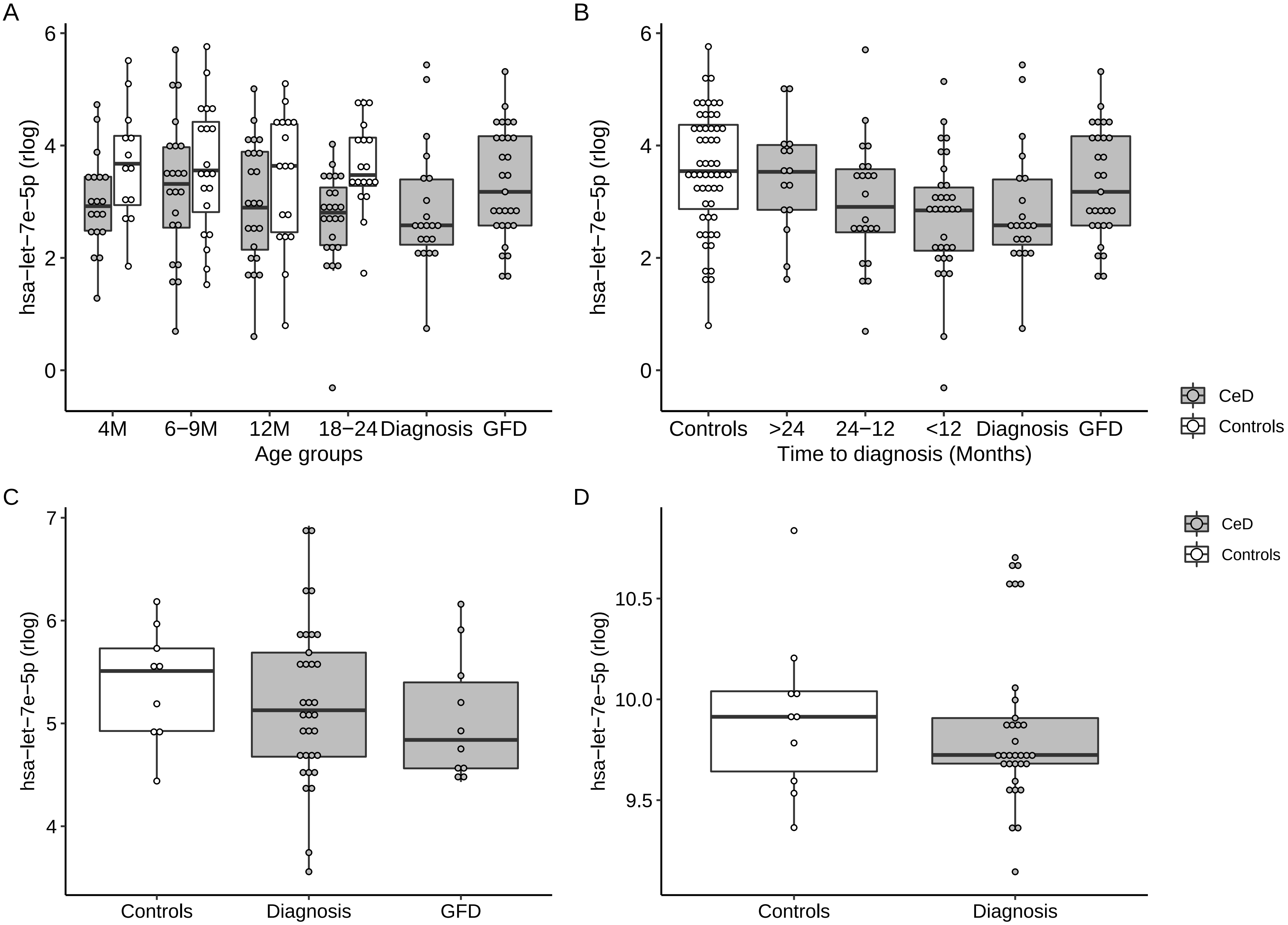

A
hsa−let−7e−5p (rlog)
0
2
4
6
4M
6−9M
12M
18−24
Diagnosis
GFD
Age groups
B
hsa−let−7e−5p (rlog)
0
2
4
6
Controls
>24
24−12
<12
Diagnosis
GFD
Time to diagnosis (Months)
CeD
Controls
C
hsa−let−7e−5p (rlog)
4
5
6
7
Controls
Diagnosis
GFD
D
hsa−let−7e−5p (rlog)
9.5
10.0
10.5
Controls
Diagnosis
CeD
Controls

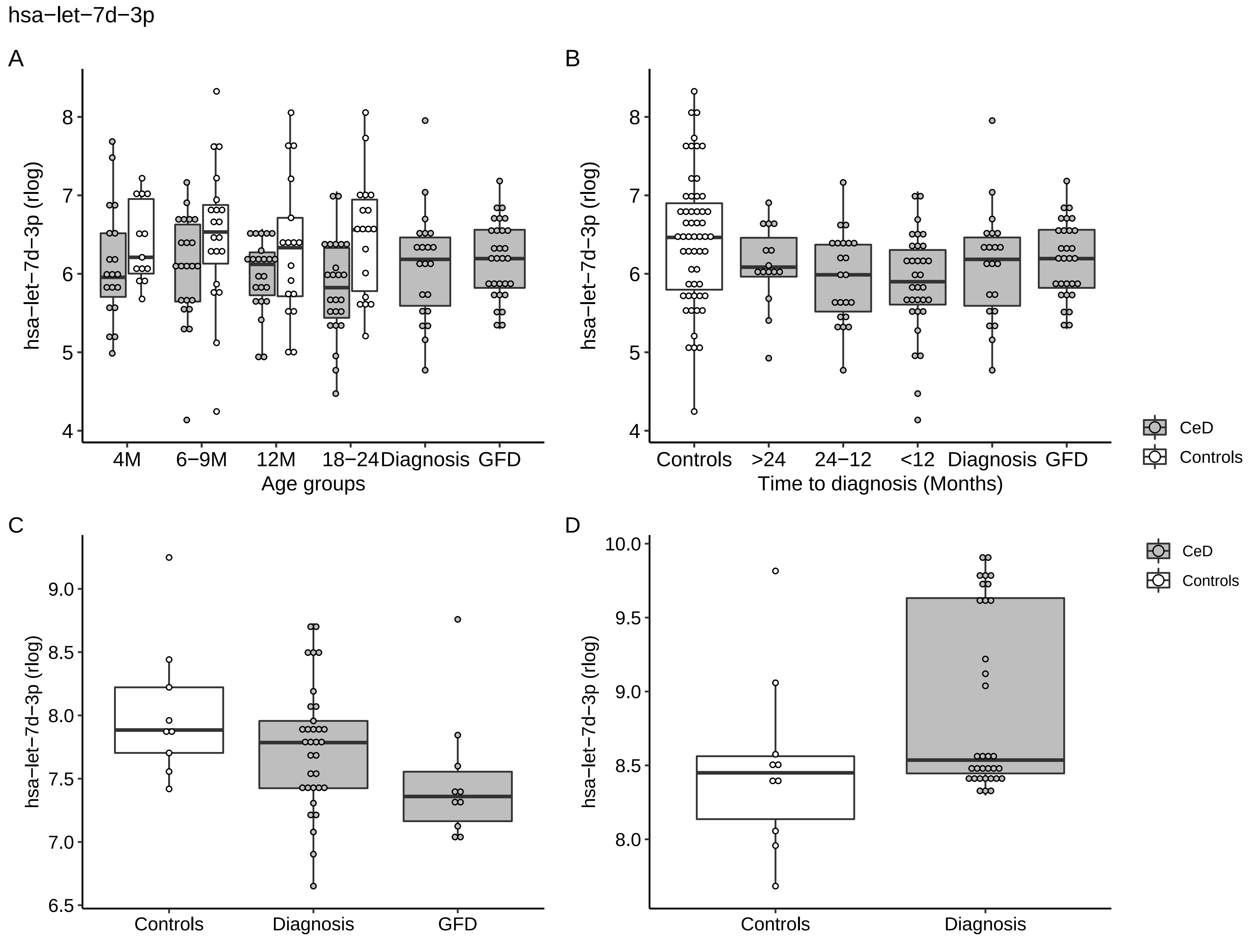
hsa−let−7d−3p
A
hsa−let−7d−3p (rlog)
4M
6−9M
12M
18−24
Diagnosis
GFD
Age groups
B
Controls
>24
24−12
<12
Time to diagnosis (Months)
CeD
Controls
C
D

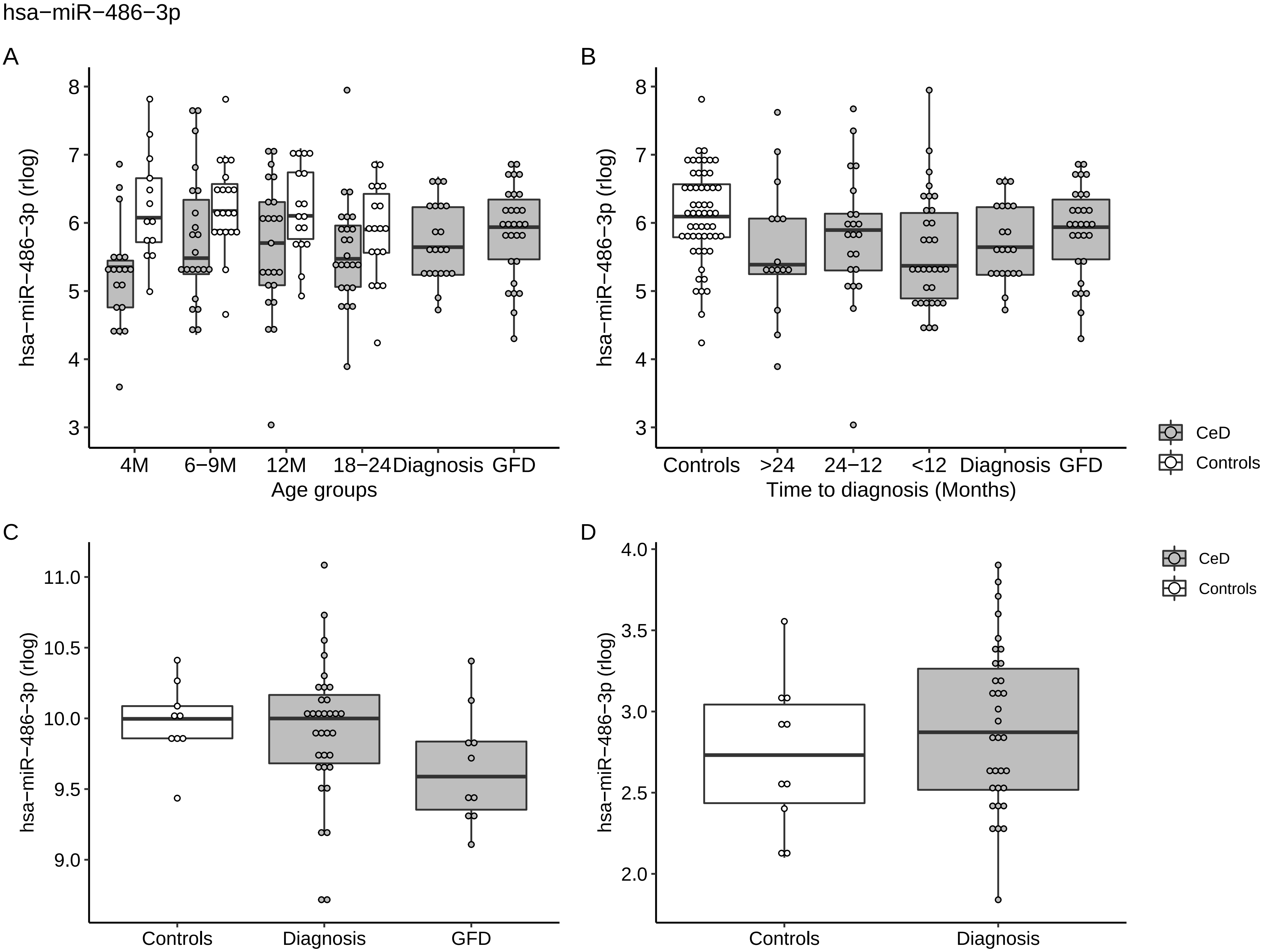
hsa−miR−486−3p
A
hsa−miR−486−3p (rlog)
8
7
6
5
4
3
4M
6−9M
12M
18−24
Diagnosis
GFD
Age groups
B
hsa−miR−486−3p (rlog)
Controls
>24
24−12
<12
Diagnosis
GFD
Time to diagnosis (Months)
CeD
Controls
C
hsa−miR−486−3p (rlog)
11.0
10.5
10.0
9.5
9.0
Controls
Diagnosis
GFD
D
hsa−miR−486−3p (rlog)
4.0
3.5
3.0
2.5
2.0
Controls
Diagnosis
CeD
Controls

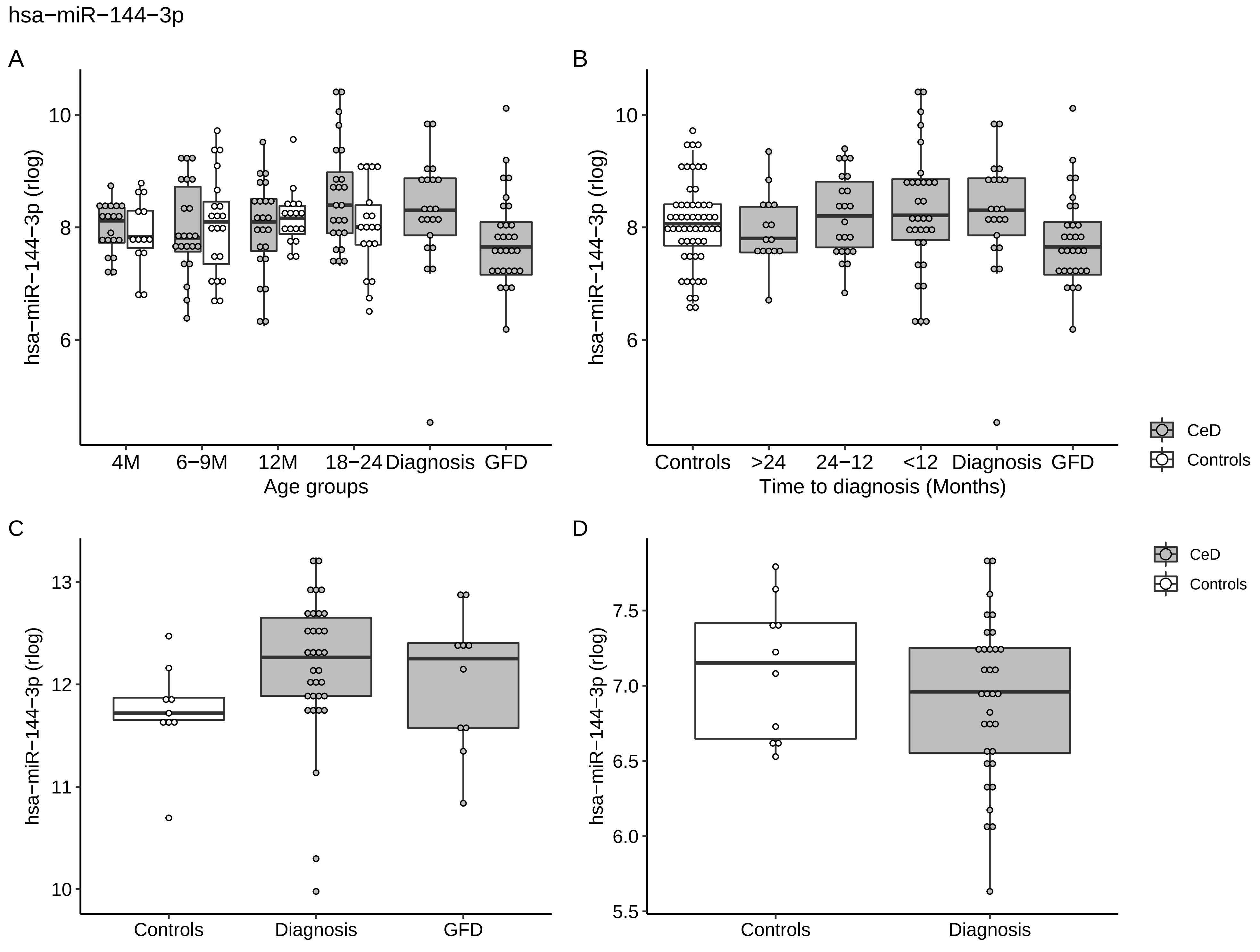
hsa−miR−144−3p
A
B
C
D
hsa−miR−144−3p (rlog)
Age groups
4M
6−9M
12M
18−24
Diagnosis
GFD
Time to diagnosis (Months)
Controls
>24
24−12
<12
CeD
Controls

hsa−miR−500a−3p

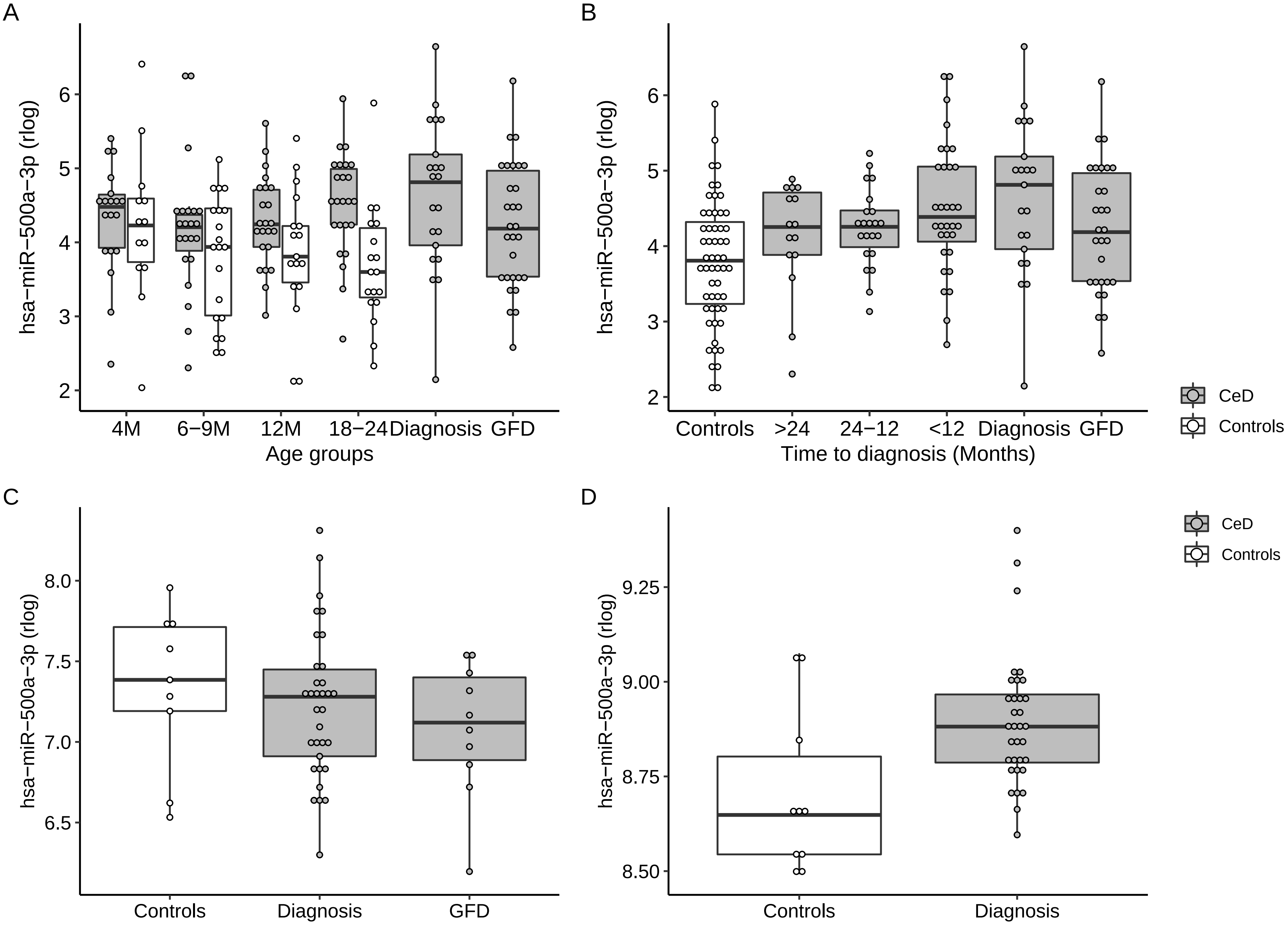

hsa−miR−374a−5p

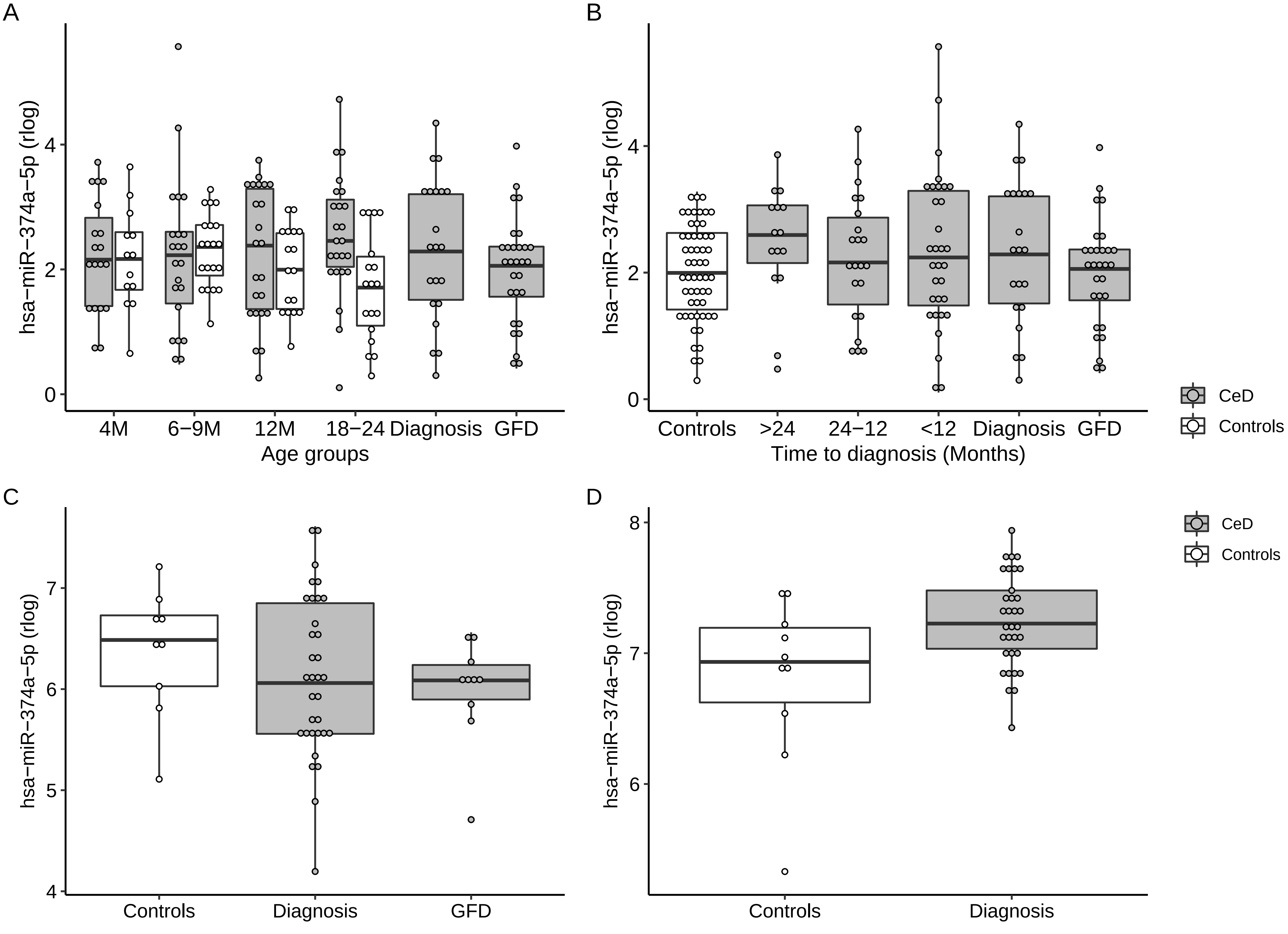

hsa−miR−21−3p

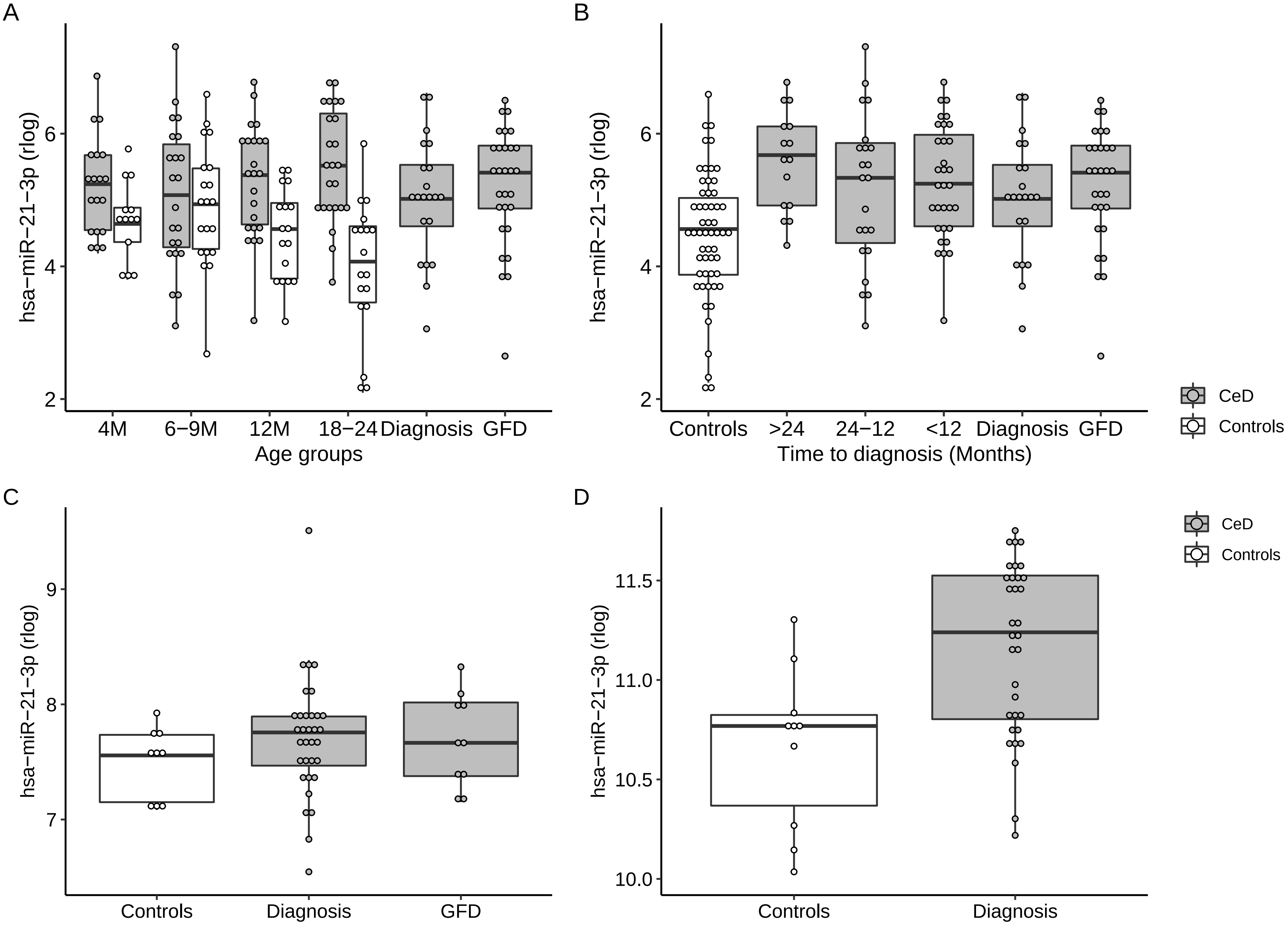
A
hsa−miR−21−3p (rlog)
2
4
6
4M
6−9M
12M
18−24
Diagnosis
GFD
Age groups
B
hsa−miR−21−3p (rlog)
2
4
6
Controls
>24
24−12
<12
Diagnosis
GFD
Time to diagnosis (Months)
CeD
Controls
C
hsa−miR−21−3p (rlog)
7
8
9
Controls
Diagnosis
GFD
D
hsa−miR−21−3p (rlog)
10.0
10.5
11.0
11.5
Controls
Diagnosis
CeD
Controls
